# Whole-exome sequencing with targeted analysis and epilepsy after acute symptomatic neonatal seizures

**DOI:** 10.1038/s41390-021-01509-3

**Published:** 2021-04-12

**Authors:** Adam L. Numis, Gilberto da Gente, Elliott H. Sherr, Hannah C. Glass

**Affiliations:** 1 Department of Neurology, University of California San Francisco, San Francisco, CA USA; 2 Department of Pediatrics, UCSF Benioff Children’s Hospital, University of California San Francisco, San Francisco, CA USA; 3 Department of Epidemiology & Biostatistics, University of California San Francisco, San Francisco, CA USA

## Abstract

**Background:**

The contribution of pathogenic gene variants with development of epilepsy after acute symptomatic neonatal seizures is not known.

**Methods:**

Case–control study of 20 trios in children with a history of acute symptomatic neonatal seizures: 10 with and 10 without post-neonatal epilepsy. We performed whole-exome sequencing (WES) and identified pathogenic de novo, transmitted, and non-transmitted variants from established and candidate epilepsy association genes and correlated prevalence of these variants with epilepsy outcomes. We performed a sensitivity analysis with genes associated with coronary artery disease (CAD). We analyzed variants throughout the exome to evaluate for differential enrichment of functional properties using exploratory KEGG searches.

**Results:**

Querying 200 established and candidate epilepsy genes, pathogenic variants were identified in 5 children with post-neonatal epilepsy yet in only 1 child without subsequent epilepsy. There was no difference in the number of trios with non-transmitted pathogenic variants in epilepsy or CAD genes. An exploratory KEGG analysis demonstrated a relative enrichment in cell death pathways in children without subsequent epilepsy.

**Conclusions:**

In this pilot study, children with epilepsy after acute symptomatic neonatal seizures had a higher prevalence of coding variants with a targeted epilepsy gene sequencing analysis compared to those patients without subsequent epilepsy.

**Impact:**

- We performed whole-exome sequencing (WES) in 20 trios, including 10 children with epilepsy and 10 without epilepsy, both after acute symptomatic neonatal seizures.
- Children with post-neonatal epilepsy had a higher burden of pathogenic variants in epilepsy-associated genes compared to those without post-neonatal epilepsy.
- Future studies evaluating this association may lead to a better understanding of the risk of epilepsy after acute symptomatic neonatal seizures and elucidate molecular pathways that are dysregulated after brain injury and implicated in epileptogenesis.

## Introduction

Neonatal seizures due to brain injury (acute symptomatic seizures) are typically self-limited in the neonatal period, but as many as 25% of survivors will later develop recurrent unprovoked seizures (epilepsy) and approximately 10% of survivors are diagnosed with infantile spasms (IS).^1–4^ Known risk factors for epilepsy after acute symptomatic seizures include severity of neonatal encephalopathy, low birth weight, low blood pH on the first day of life, abnormal neuroimaging, multifocal (versus focal) seizures, >1 medicine to control neonatal seizures, status epilepticus, persistently abnormal electroencephalogram (EEG) background, and seizure spread to the contralateral hemisphere.^5–7^ Yet, not every neonate with risk factors will develop epilepsy, and the most vulnerable children cannot be prospectively identified. Furthermore, little is known about the pathophysiologic mechanisms of epileptogenesis following neonatal brain injury.

Next-generation sequencing has transformed our understanding of epilepsy genetics; hundreds of genes have potential association with recurrent seizures. Pathogenic variants can result in syndromes with epilepsy as the core symptom (e.g., *SCN1A* and Dravet syndrome), cause brain malformations and other physical or developmental anomalies associated with epilepsy (e.g., *TSC1*/*TSC2* and tuberous sclerosis complex), or alter inherent seizure susceptibility (e.g., *CACNA1H*).^8,9^ Whereas the utility of whole-exome sequencing (WES) with targeted gene analysis has been important for establishing diagnosis and prognosis of severe early-onset epileptic encephalopathies,^10,11^ the contribution of gene variants to epileptogenesis after acute symptomatic neonatal seizures is not known, but we hypothesize that genetic risk factors play a role.

In this pilot case–control study, we examined WES in family trios from children affected by acute symptomatic neonatal seizures with and without post-neonatal epilepsy to determine whether there is an increased incidence of de novo and inherited loss-of-function mutations in known genes associated with epilepsy versus genes in an unrelated group of disorders, in this case those associated with coronary artery disease (CAD). We evaluated remaining WES variants using pathway analysis to evaluate differential enrichment of functional biologic processes in those with and without post-neonatal epilepsy. We hypothesize that de novo and inherited mutations in established and candidate genes that may alter risk of epilepsy after acute symptomatic neonatal seizures have a higher prevalence in children with epilepsy after acute symptomatic seizures compared to children without post-neonatal epilepsy.

## Methods

This was a case–control study of WES family trios including a proband with a history of acute symptomatic seizures and aged at least 2 years and their biological mother and father. Ten children who developed epilepsy in childhood (cases) were compared with 10 children who remained free from epilepsy until at least 2 years of age (controls). All participants were recruited from the University of California, San Francisco (UCSF) Benioff Children’s Hospital. First, we recruited among participants enrolled in the Neonatal Seizure Registry at UCSF (NCT02789176), a multicenter prospective cohort study of neonates with acute symptomatic seizures.^12^ Additional cases of children with epilepsy after neonatal acute symptomatic seizures were enrolled from a clinic-based convenience sample of patients seen at the UCSF Pediatric Epilepsy Center of Excellence or the Neuro-Intensive Care Nursery follow-up program from 3/2019 to 5/2019. We included children with a history of acute symptomatic seizures with onset <44 weeks postmenstrual age. Seizure etiology included, but was not limited to, hypoxic–ischemic encephalopathy (HIE), ischemic stroke, or cerebral hemorrhage.^13^ We excluded children with risk for epilepsy *independent of* seizures and underlying brain injury (including, but not limited, to inborn errors of metabolism or brain malformations), as well as transient cause for seizures (e.g., mild hypoglycemia, hyponatremia, hypocalcemia with normal neuroimaging), and neonatal-onset epilepsy syndromes. The study protocol was approved by the UCSF Committee on Human Research and both biologic parents of each child provided written informed consent.

### Clinical data abstraction

Hospital records were reviewed to determine demographic data, seizure etiology, continuous video EEG results, neuroimaging results, and antiseizure medication (ASM) use. Neonatal seizure etiology was determined by a pediatric neurologist (A.L.N. and H.C.G.) after reviewing clinical and imaging records and was classified as follows: HIE, ischemic stroke, intracranial hemorrhage, infection, hypoglycemia, or other. Seizure classification (clinical, electroclinical, or electrographic only) and burden in the Neuro-Intensive Care Nursery was determined by a review of the clinical report by a board-certified clinical neurophysiologist (A.L.N.). A neonate was considered to have seizures without EEG confirmation (i.e., clinical seizures) if they had paroxysmal events with a semiology consistent with neonatal seizures warranting treatment with an ASM before EEG monitoring was initiated. Subclinical or electrographic only seizures were defined as sudden, abnormal EEG events with repetitive and evolving pattern with amplitude ≥2 μV and duration ≥10 s without a clinical correlate.^14^ Seizure burden was defined as follows: (1) no electrographic seizures, (2) rare electrographic seizures (less than seven), (3) many isolated electrographic seizures (seven or more), (4) frequent recurrent seizures not meeting criteria for status epilepticus, and (5) status epilepticus.^15^

Outpatient follow-up records from primary care, neurology clinic, subspecialty visits, and the Intensive Care Nursery Follow Up Program were reviewed to determine the presence of seizures after the neonatal period. The primary outcome, post-neonatal epilepsy, was defined per International League Against Epilepsy (ILAE) 2014 criteria.^16^ IS was defined according to ILAE criteria as seizures characterized by “epileptic spasms… a sudden flexion, extension, or mixed extension–flexion of predominantly proximal and truncal muscles” occurring in clusters and during infancy. Intractable epilepsy was defined as failure of two appropriate ASMs.

### Genetic sampling, whole-exome capture, and sequencing

Participants were contacted for participation from 5/2019 to 8/2019. After consenting to participate in this study, families were mailed validated self-collection and assisted saliva-based collection kits (DNA Genotek OGR-500 and OGR-575). Samples was returned and stored at 4 °C until processing at the UCSF Institute for Human Genetics Genomics Core. DNA was isolated using the Qiagen Gentra Puregene system. DNA was fragmented using a Covaris LE220 to a size range of ~350 bases and assembled into a library constructed with unique dual indexes compatible with NovaSeq. As previously described, exome sequencing was performed using the NimbleGen Human SeqCap EZ Exome (v3.0) Kit according to the manufacturer’s protocol in 12/2019.^17^ Libraries were pooled into a capture reaction that contains biotinylated oligonucleotide probes to target specific regions of interest. The biotinylated probe/target hybrids were pulled down by streptavidin-coated magnetic beads to obtain libraries highly enriched for the target regions. WES was performed using the Illumina NovaSeq 6000. Sequencing data were transferred using gzipped fastq format for analysis.

### Exome data analysis

In our primary analysis, we restricted WES data to 200 genes found in commercially available epilepsy gene panels (GeneDx “Comprehensive epilepsy panel,” Gaithersburg, MD; and, Invitae “Epilepsy Panel,” San Francisco, CA; Table S1). These panels include the eight genes curated by the Clin Gene Epilepsy Gene Curation Expert Panel in 2018 as having definitive or strong evidence of an epilepsy association (*ALG13*, *CHD2*, *DNM1*, *KCNA2*, *KCNQ2*, *KCNT1*, *SCN8A*, and *STXBP1*) as well as eight genes with limited or disputed evidence (*CACNA1H*, *CACNB4*, *EFHC1*, *GRIN2D*, *MAGI2*, *RYR3*, as well as *SCN9A*, and *SRPX2*).^18^ The remaining genes in these panels have varying, at times contradictory, levels of evidence for an epilepsy association; however, we included these genes in our analyses given our exploratory aim and hypothesis that established and candidate epilepsy *association* genes, more so than genes associated with an epilepsy syndrome (i.e., *SCN1A*, *KCNQ2/3*), will increase the risk of epilepsy after neonatal acute symptomatic seizures. As a sensitivity analysis, we restricted WES data to a subset of 89 non-overlapping genes associated with CAD as previously described.^17^ In a secondary analysis, we analyzed the complete WES dataset.

Our analytic pipeline followed “The Broad Institute’s Best Practices” guidelines for discovering putative variants and utilizes the Genome Analysis Toolkit (software version 2014.23.1.7-10) in combination with BWA-mem, Picard Tools, and SAM Tools as previously described.^19^ In brief, after aligning the DNA read sequences to the GRCh37 reference build using BWA-mem, Picard Tools were used to identify and remove PCR duplicates, add read group information, and sort alignment files using modules Mark Duplicates, SortSam, and AddOrReplaceReadGroups, respectively. All variants were compared to parental samples to determine whether they were de novo or inherited from the biological mother or father.

For all analyses, variants were required to be within the transcript region (identified as a missense/nonsense single-nucleotide variant or out-of-frame small insertion or deletion (indel)) or within 3 base pairs of a splice site, be below a population frequency of 0.1% (as determined by 1000 Genomes and the Exome Variant Server 6500), a CADD score of >20, and genotype quality (GQ) of >50. For de novo analyses, all variants had a minimum of 10 reads with at least 3 showing the alternate variant in addition to an allelic balance >0.25. In targeted gene sets, allelic balance requirement was lowered to 0.1. In both analyses, parents were required to have a minimum GQ of 50 with no reads showing the alternate variant. For inheritance analysis in targeted gene panels, variants were separated into subgroups of transmitted (passed from parent to child) or non-transmitted (not passed from parent to child).

### Variant classification

Each variant was annotated against a reference transcript. In silico modeling with Polyphen-2 (HumDiv and HumVar) was used to assess protein structure/function and evolutionary conservation. Variants were classified as “pathogenic,” “likely pathogenic,” “benign,” “likely benign,” or a “variant of uncertain significance” (VUS), according to the American College of Medical Genetics and Genomics (ACMG) guidelines.^20^ Pathogenic variants were confirmed with visual inspection in IGV. The biological relevance of all affected variants was evaluated using the Online Mendelian Inheritance in Man database, ClinVar, gnomAD, and Uniprot.^21–24^

### KEGG pathway enrichment analysis

De novo pathogenic, likely pathogenic, and VUS identified in the complete WES dataset were analyzed for functional properties using Kyoto Encyclopedia of Genes and Genomes (KEGG) searches.^25–27^ Given the limitations of power with sample size, we limited pathway analysis to KEGG orthology and excluded categorization of human disease (09160) and organismal systems (09150) apart from the nervous system (09156).

### Statistical analyses

Statistical analyses were performed using the Stata 15.1 software. Chi-square test was used to compare categorical variables and *t* test for continuous variables. Significance was determined as *p* < 0.05.

## Results

We conducted WES in 20 trios, of whom 10 probands developed post-neonatal epilepsy at a median age of 16 months (interquartile range (IQR) 5–24 months). Among 26 potential participants enrolled in the Neonatal Seizure Registry at UCSF, 2 of 3 (67%) with post-neonatal epilepsy and 10 of 23 (44%) without post-neonatal epilepsy enrolled in this investigation.^28^ The remaining cases of children with post-neonatal epilepsy were identified in a clinic-based convenience sample, with 8 of 10 (80%) consecutive patients enrolling. Median age of follow-up in children without epilepsy at the time of enrollment into this study was 3.2 years (IQR 2.5–3.8 years), with 70% of children having >3 years of follow-up and no child having >5 years of follow-up. The median age at the time of WES was 3.5 years (IQR 2.4–17.8 years). Children with post-neonatal did not differ from those without epilepsy with regards to duration of follow-up at the time of enrollment (*p* = 0.51) or at the age when next-generation sequencing was performed (*p* = 0.50).

In children with post-neonatal epilepsy, five were diagnosed with IS, of whom two had HIE, one had ischemic stroke, one had intracranial hemorrhage, and one had infection as cause of their acute symptomatic neonatal seizures. Children with and without post-neonatal epilepsy did not differ by sex, mode of delivery, gestational age, birth weight, neonatal seizure burden, or seizure treatment in the neonatal period (Table 1). The underlying etiologies for neonatal seizures were similar between groups, with HIE, ischemic stroke, and intracranial hemorrhage accounting for the majority in children with and without post-neonatal epilepsy.

**Table 1:** Clinical characteristics of 10 children with and 10 without post-neonatal epilepsy after acute symptomatic neonatal seizures.

	Epilepsy (*n* = 10)	No epilepsy (*n* = 10)	Total	*p* value
**Male**, *n* (%)	6 (60)	3 (30)	9 (45)	0.18
**Race**, *n* (%)				
Caucasian	7 (70)	5 (50)	12 (60)	0.09
African-American	0	0	0	
Asian	1 (10)	5 (50)	6 (30)	
Multiracial/other	2 (20)	0	2 (10)	
**Ethnicity (Hispanic)**, *n* (%)	2 (20)	0	2 (10)	0.14
**Term (>37 weeks)**, *n* (%)	6 (60)	8 (80)	14 (70)	0.33
**Birth weight in kg**, median (IQR)	2.8 (1.9–3.3)	3.1 (2.9–3.4)	3.1 (2.5–3.3)	0.53
**Apgar 5 min**, median (IQR)	9 (8–9)	9 (6–9)	9 (6–9)	0.45
**Seizure onset after birth in hours**, median (IQR)	24 (12–60)	22 (15–33)	23(12–48)	0.57
**Seizure etiology**, *n* (%)				
HIE/NE	2 (20)	3 (30)	5 (25)	0.77
Ischemic stroke	2 (20)	2 (20)	4 (20)	
Intracranial hemorrhage	1 (10)	3 (30)	4 (20)	
Infection	2 (20)	1 (10)	3 (15)	
Hypoglycemia	2 (20)	0	2 (10)	
Unknown	1 (10)	1 (10)	2 (10)	
**Seizure type**, *n* (%)				
Electroclinical	6 (60)	8 (80)	14 (70)	0.35
Clinical only	3 (20)	1 (10)	3 (15)	
Electrographic only	0	1 (20)	2 (10)	
Documentation inadequate to identify	1 (10)	0	1 (5)	
**Seizure semiology**, *n* (%)				
Subtle	2 (20)	0	2 (10)	0.30
Clonic	4 (40)	7 (70)	11 (55)	
Mixed subtle and clonic	1 (10)	1 (10)	2 (10)	
Subclinical	0	1 (10)	1 (5)	
Documentation inadequate to identify	3 (30)	1 (10)	4 (20)	
**Seizure burden**, *n* (%)				
Rare (<7)	5 (50)	4 (40)	9 (45)	0.56
Many isolated (≥7)	1 (10)	2 (20)	3 (15)	
Frequent recurrent	0	1 (10)	1 (5)	
Status epilepticus	2 (20)	3 (30)	5 (25)	
Documentation inadequate to identify	2 (20)	0	2 (10)	
**Medications administered in the neonatal period**, n (%)				
Intermittent benzo	2 (20)	3 (30)	5 (25)	0.87
Phenobarbital	10 (100)	6 (60)	16 (80)	
Fosphenytoin	4 (40)	5 (50)	9 (45)	
Levetiracetam	4 (40)	3 (30)	7 (35)	
Benzo infusion	1 (10)	1 (10)	2 (10)	
**Age at initial hospital discharge in days**, median (IQR)	30 (18–42)	11 (6–13)	13 (7–42)	0.18

*ASM* antiseizure medication, *benzo* benzodiazepine, *EEG* electroencephalogram, *HIE/NE* hypoxic–ischemic encephalopathy/neonatal encephalopathy, *IQR* interquartile range.

### Targeted gene analysis (epilepsy gene panel)

Among the 200 established and candidate epilepsy association genes, we identified 29 variants: 4 de novo variants in 3 genes and 25 inherited variants in 23 genes. Six (21%) of the 29 variants were classified as pathogenic or likely pathogenic in 6 participants (Table 2), 17 (58%) as benign or likely benign, and the remaining 6 (21%) as VUS (Table S2). All inherited variants were also found in a parent without a history of epilepsy. There was no difference in variant type (missense, nonsense, frameshift, splice site) between children with and without post-neonatal epilepsy.

**Table 2:** Pathogenic variants in established and candidate epilepsy genes.

Case	Gene	Transcript reference	Type	Class	AA change	cDNA variant^a^	CADD Phred	Polyphen-2 scores^b^	Post-neonatal epilepsy	Infantile spasms
1	*CASK*	ENST00000442742	Inherited (mat)	Missense	p.R618K	c.1853G>A	21.4	1.0/1.0	Yes	No
2	*MAGI2*	ENST00000522391	De novo	Splice	—	g.77694888T>G	21.1	—	Yes	Yes
3	*PRRT2*	ENST00000567659	Inherited (mat)	Missense	p.P362T	c.1084C>A	24.4	0.99/0.94	Yes	Yes
4	*RBFOX3*	ENST00000415831	De novo	Missense	p.A207V	c.620C>T	34.0	0.98/0.67	Yes	Yes
5	*KCNT1*	ENST00000490355	Inherited (mat)	Missense	p.V296M	c.886G>A	27.6	1.0/1.0	Yes	No
6	*CPA6*	ENST00000297770	Inherited (mat)	Nonsense	p.R311*	c.931C>T	46.0	—	No	—

*AA* amino acid, *mat* maternal, *pat* paternal.

^a^HGVS notation and GRCh37 genome reference.

^b^Polphen-2 scores presented as HumDiv/HumVar.

The six pathogenic/likely pathogenic variants in epilepsy-associated genes were more common among children with post-neonatal epilepsy (5/10 children, 50%) as compared to those without (1/10, 10%). Children with epilepsy had 9.0 times the odds of having a pathogenic/likely pathogenic variant compared to those without post-neonatal epilepsy (95% confidence interval (CI) 0.6–472, *p* = 0.05). In contrast, in a similar analysis using known CAD genes, one child with post-neonatal epilepsy and one child without epilepsy had a pathogenic/likely pathogenic variant identified (odds ratio (OR): 1.0, 95% CI: 0.01–87, *p* = 1.0, Table S3). Similarly, there was no difference in the number of families with non-transmitted pathogenic or likely pathogenic variants in epilepsy or CAD genes (in each analysis, one parent of a child with epilepsy and no parent of a child without epilepsy had pathogenic variants identified; Table S4).

Among children with post-neonatal epilepsy, two pathogenic/likely pathogenic variants and two VUS were in candidate genes that may alter susceptibility to epilepsy (*CACNA1H*, *CASK*, *RBFOX3*, and *RYR3*),^9,29–31^ and three pathogenic/likely pathogenic variants were in established and candidate genes that are associated with epilepsy syndromes with incomplete penetrance and variable expressivity (*KCNT1*, *MAGI2*, and *PRRT2*).^32–34^ Three of the five children with IS had pathogenic/likely pathogenic variants. Among the children without post-neonatal epilepsy, the one pathogenic variant identified was in *CPA6*, a candidate gene that may result in epilepsy with onset through 12–18 years of age, outside the window of follow-up in this cohort.^32,35–37^

### WES analysis with KEGG orthology

**Fig. 1:**
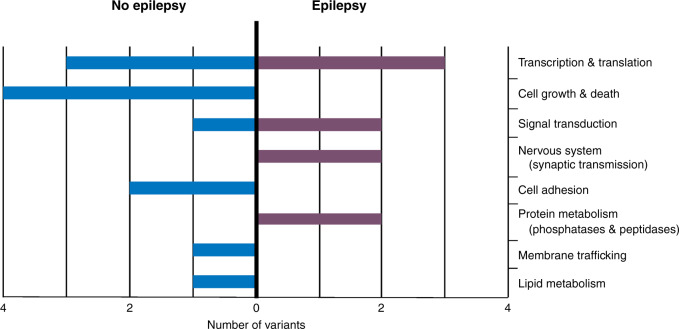
KEGG orthology categorization of pathogenic variants on whole exome.

Seventeen de novo pathogenic/likely pathogenic variants or VUS were found in genes without a known association with epilepsy (Table 3). Seven of the variants were found in 6 children with post-neonatal epilepsy and 10 of the variants were found in 6 children without post-neonatal epilepsy (OR 1.0, 95% CI 0.11–8.4, *p* = 1.0). There was no difference between groups with respect to variant type (missense, nonsense, frameshift, splice site). An exploratory KEGG orthology analysis demonstrated that children who developed post-neonatal epilepsy had a relative enrichment in variants associated with the nervous system, including synaptic transmission, and those without epilepsy had a relative enrichment of variants associated with cell growth and death, in particular the ubiquitin system (Fig. 1).

**Table 3:** De novo variants identified on whole-exome sequencing.

Case	Gene	Transcript reference	Mutation type	AA change^a^	cDNA variant^a^	CADD Phred	Polyphen-2 scores^b^	ACMG pathogenicity	Post-neonatal epilepsy
2	*ENOX1*	ENST00000540032	Missense	p.R111C	c.331C>T	31.0	1.0/0.99	Likely pathogenic	Yes
	*PTPN14*	ENST00000366956	Missense	p.E712D	c.2136G>C	23.1	0.99/0.97	Likely pathogenic	
3	*ATF6B*	ENST00000375201	Missense	p.G634E	c.1901G>A	25.5	0.98/0.70	Likely pathogenic	Yes
4	*SNRPB*	ENST00000381342	Missense	p.R18K	c.53G>A	23.7	0.84/0.49	Likely pathogenic	Yes
7	*MIF4GD*	ENST00000577542﻿	Nonsense	p.E201*	c.601G>T	42.0	—	Pathogenic	Yes
9	*GNB3*	ENST00000229264	Missense	p.R8C	c.22C>T	24.9	0.95/0.52	Likely pathogenic	Yes
10	*CAPN7*	ENST00000253693﻿	Missense	p.V435I	c.1303G>A	25.6	0.99/0.93	Likely pathogenic	Yes
8	*DHRS11*	ENST00000611337	Missense	p.V12G	c.35T>G	28.3	0.99/0.94	Likely pathogenic	No
	*FAM186A*	ENST00000327337	Frameshift	p.Q1609HfsTer47	c.4826_4827insCA	22.6	—	Pathogenic	
11	*HECTD4*	ENST00000550722	Splice	—	g.112819897C>A	20.2	—	Likely pathogenic	No
	*PCBP2*	ENST00000549863	Splice	—	g.53845910G>A	22.4	—	VUS	
	*VPS37A*	ENST00000324849	Missense	p.S10R	c.30C>G	21.8	0.97/0.41	Likely pathogenic	
12	*ELF1*	ENST00000239882	Missense	p.V142I	c.424G>A	23.3	0.99/0.99	VUS	No
13	*CSTF2T*	ENST00000331173	Missense	p.E26K	c.76G>A	32.0	0.99/0.99	Likely pathogenic	No
	*UBXN11*	ENST00000357089	Frameshift	p.C453Sfs*?	c.1357_1358insCCCGGCCCCA	22.2	—	Pathogenic	
14	*DCHS2*	ENST00000357232	Nonsense	p.E270*	c.808G>T	25.8	—	Pathogenic	No
15	*OSBP*	ENST00000263847	Missense	p.D461H	c.1381G>C	31.0	1.0/0.99	Likely pathogenic	No

*AA* amino acid, *ACMG* American College of Medical Genetics, *VUS* variant of uncertain significance.

^a^HGVS notation and GRCh37 genome reference.

^b^Polphen-2 scores presented as HumDiv/HumVar.

## Discussion

In this pilot case–control study of 20 trios of children with a history of acute symptomatic seizures with and without subsequent epilepsy, WES with targeted analysis of established and candidate epilepsy-associated genes identified six de novo or inherited pathogenic/likely pathogenic variants in six children. Children with epilepsy had increased odds of having a pathogenic/likely pathogenic variant compared to those without post-neonatal epilepsy. There was no difference in the odds of having a pathogenic/likely pathogenic variant in CAD genes, or a difference in the odds non-transmitted variants in epilepsy or CAD genes between groups, suggesting that the findings are related to the development of epilepsy. We propose that the “double hit” of a pathogenic/likely pathogenic variant in an established or candidate epilepsy association gene and acute symptomatic seizures in the neonatal period increases risk of epilepsy more than acute symptomatic seizures alone. Our findings add to the growing literature about the use of genetic testing to understand epilepsy. Targeted gene panels and WES are considered important for defining diagnosis and understanding prognosis of a wide range of non-acquired epilepsies from severe early-onset epileptic encephalopathies to focal epilepsies in adulthood.^10,11,38^ If replicated in a larger cohort, our findings suggest that genetic testing may also enable us to better predict the subsequent risk of epilepsy after acute neonatal symptomatic seizures.

Identification of variants in established and candidate epilepsy association genes may also inform ASM management. For example, in our cohort, two children with intractable post-neonatal epilepsy had variants in the *CASK* and *CACNA1H* genes. The *CASK* gene encodes the protein calcium/calmodulin-dependent serine protein kinase regulating alpha-amino-3-hydroxy-5-methyl-4-isoxazolepropionic acid (AMPA) receptor trafficking.^39,40^ Perampanel is a selective, non-competitive AMPA agonist and with potential to rescue CASK-mediated disruption. The *CACNA1H* gene encodes a subunit of the voltage-dependent calcium channel complex. CACNA1H-associated epilepsy has demonstrated responsiveness to lamotrigine or ethosuximide therapy.^9,41^ In our cohort, children with variants in these genes have at least weekly seizures, have failed three or more ASMs to control their seizures, and had not yet trialed possible precision medicine therapies. Targeted gene analysis may inform providers regarding ASM selection, providing an individualized approach to epilepsy management.

WES with gene set enrichment analysis to compile an individual’s genetic variant burden in pathways that are over- or under-represented can inform exploration of molecular processes that may facilitate or suppress epileptogenesis.^42,43^ In our secondary analysis, WES analysis demonstrated a similar number of de novo variants throughout the exome among those with and without epilepsy after acute symptomatic neonatal seizures. Exploratory KEGG orthology analysis revealed differences in the relative enrichment of variants in key molecular processes between groups. Notably, those with post-neonatal epilepsy had enrichment of variants associated with synaptic transmission while those without post-neonatal epilepsy had enrichment of variants in cell growth and death pathways, in particular the ubiquitin pathway. This finding is compelling, given that epilepsy is caused by an imbalance between neuronal excitation and inhibition and alterations in synaptic transmission contribute to the disease process, although the impact of the pathogenic variants in our cohort is not known. Ubiquitin is a regulatory protein associated with neurologic disease through its effects on neural development and maintenance via post-translational modifications and resultant protein degradation.^44^ Levels of the brain-enriched enzyme ubiquitin C-terminal hydrolase-L1 can predict neuronal injury after traumatic brain injury, ischemic brain injury, and neonatal HIE.^45,46^ These data, while under-powered, can inform future evaluation of single-nucleotide polymorphisms that alter function within these pathways with the aim of improving our understanding mechanism of epileptogenesis after brain injury.^47^

This single-center study has limitations. First, the small sample size limits immediate generalizability. Second, although we only studied children whose epilepsy onset was before age 2 years, the duration follow-up for children without epilepsy was relatively short (2–5 years), and so these children may yet develop epilepsy.^48,49^ For example, the pathogenic variant in the *CPA6* gene identified in our control group (children without epilepsy through at least 2 years of age) can increase epilepsy susceptibility into late childhood;^38,39,41^ longer duration of follow-up could result in re-classification of this proband. Third, gene sequencing has inherent limitations in predicting the consequences of DNA variants on protein function. While our methods use ACMG criteria for variant classification so as to use best practices and limit future re-categorization, these are only applicable to the six genes with definitive or strong evidence for an epilepsy association.^18,20^ By applying these methods to candidate epilepsy genes, we may be incorrectly categorizing variants as “pathogenic” or “likely pathogenic” for an epilepsy association and biasing our results.

If the role of targeted gene analysis to predict post-neonatal epilepsy after acute symptomatic seizures is supported with future studies, epilepsy gene panels could enhance established prediction paradigms that currently incorporate clinical, EEG, and radiologic data, allowing for improved counseling of providers and families.^6,7,13,48,49^ Future investigations of genetic sequencing in larger cohorts, perhaps leveraging existing databases of neonates with acute symptomatic seizures with longer follow-up duration, may test this hypothesis with multivariate modeling of genetic data along with known risk factors of post-neonatal epilepsy. WES in larger cohorts also will allow robust bioinformatic analysis and hierarchical clustering to visualize and explore functional pathways associated with epilepsy after acute symptomatic seizures.^50,51^

## Supplementary information

Supplementary Materials
