# Supplementary material for: Whole-exome sequencing with targeted analysis and epilepsy after acute symptomatic neonatal seizures
Source: Pediatr Res. 2021 Apr 12;91(4):896–902. doi: 10.1038/s41390-021-01509-3 (PMC9064802; doi:10.1038/s41390-021-01509-3)

**Supplementary Materials:**

**Page**

**Supplementary Table 1:** Two hundred established and candidate epilepsy genes

included in the targeted exome analysis …………... 2

**Supplementary Table 2:** Variants of uncertain significance in established and candidate epilepsy genes ………….. 3

**Supplementary Table 3:** "Pathogenic" and "likely pathogenic" variants in coronary artery disease genes ………….. 4

**Supplementary Table 4:** Non-inherited "pathogenic" and "likely pathogenic" variants in epilepsy or coronary artery disease gene sets ………….. 5

**Supplementary Figure 1:** IGV inspection of established and candidate epilepsy genes in the proband and parents …….......... 6

**Supplementary Figure 2:** IGV inspection of *de novo* non-epilepsy gene variants identified on whole exome sequencing in the proband and parents ………….. 12

**Supplementary Table 1:** Two hundred established and candidate epilepsy genes included in the targeted exome analysis.

| *ABAT* |  | *CNTN2* |  | *GPHN* |  | *SCN1A* |  | *ROGDI* |  | *SYN1* |
| --- | --- | --- | --- | --- | --- | --- | --- | --- | --- | --- |
| *ADSL* |  | *CNTNAP2* |  | *GPHN* |  | *SCN1B* |  | *RYR3* |  | *SYNGAP1* |
| *ALDH5A1* |  | *CPA6* |  | *GRIN1* |  | *SCN2A* |  | *SATB2* |  | *SYNJ1* |
| *ALDH7A1* |  | *CSTB* |  | *GRIN2A* |  | *SCN3A* |  | *SCARB2* |  | *SZT2* |
| *ALG13* |  | *CTSD* |  | *GRIN2B* |  | *NEXMIF* |  | *TBL1XR1* |  | *TBC1D24* |
| *AMT* |  | *DEPDC5* |  | *HCN1* |  | *NGLY1* |  | *TCF4* |  | *TSC2* |
| *ARHGEF15* |  | *DIAPH1* |  | *HNRNPU* |  | *NHLRC1* |  | *TPK1* |  | *UBE3A* |
| *ARHGEF9* |  | *DNAJC5* |  | *IER3IP1* |  | *NPRL3* |  | *TSC1* |  | *WDR45* |
| *ARX* |  | *DNM1* |  | *IQSEC2* |  | *NRXN1* |  | *SCN5A* |  | *WWOX* |
| *ATP1A2* |  | *DOCK7* |  | *ITPA* |  | *PACS1* |  | *SCN8A* |  | *ZDHHC9* |
| *ATP1A3* |  | *DYRK1A* |  | *JMJD1C* |  | *PCDH19* |  | *SCN9A* |  | *ZEB2* |
| *ATP6AP2* |  | *EEF1A2* |  | *KANSL1* |  | *PIGA* |  | *SERPINI1* |  | *ASNS* |
| *ATRX* |  | *EFHC1* |  | *KCNA1* |  | *PIGG* |  | *SETD2* |  | *CHRNA7* |
| *BRAT1* |  | *EHMT1* |  | *KCNA2* |  | *PIGN* |  | *SGCE* |  | *CTNNB1* |
| *C12orf57* |  | *EPM2A* |  | *KCNB1* |  | *PIGO* |  | *SIK1* |  | *CTSF* |
| *CACNA1A* |  | *FARS2* |  | *KCNC1* |  | *PIGQ* |  | *SLC12A5* |  | *DDX3X* |
| *CACNA1H* |  | *FASN* |  | *KCND2* |  | *PIK3AP1* |  | *SLC13A5* |  | *KCNH1* |
| *CACNA2D2* |  | *FLNA* |  | *KCNH2* |  | *PLCB1* |  | *SLC19A3* |  | *KDM6A* |
| *CACNB4* |  | *FOLR1* |  | *KCNH5* |  | *PNKD* |  | *SLC25A12* |  | *KIAA2022* |
| *CARS2* |  | *FOXG1* |  | *KCNJ10* |  | *PNKP* |  | *SLC25A22* |  | *MAGI2* |
| *CASK* |  | *FRRS1L* |  | *KCNMA1* |  | *PNPO* |  | *SLC2A1* |  | *NALCN* |
| *CASR* |  | *GABBR2* |  | *KCNQ2* |  | *POLG* |  | *SLC35A2* |  | *NR2F1* |
| *CDKL5* |  | *GABRA1* |  | *KCNQ3* |  | *PPT1* |  | *SLC35A3* |  | *PIGV* |
| *CERS1* |  | *GABRB2* |  | *KCNT1* |  | *PRDM8* |  | *SLC6A1* |  | *PPP2R5D* |
| *CHD2* |  | *GABRB3* |  | *KCTD7* |  | *PRICKLE1* |  | *SLC6A8* |  |  |
| *CHRNA2* |  | *GABRD* |  | *KPNA7* |  | *PRICKLE2* |  | *SLC9A6* |  |  |
| *CHRNA4* |  | *GABRG2* |  | *LGI1* |  | *PRIMA1* |  | *SMC1A* |  |  |
| *CHRNB2* |  | *GAL* |  | *LIAS* |  | *PRRT2* |  | *SPATA5* |  |  |
| *CLCN4* |  | *GAMT* |  | *LMNB2* |  | *PTEN* |  | *SPTAN1* |  |  |
| *CLN2 (TPP1)* |  | *GATM* |  | *MBD5* |  | *PURA* |  | *SRPX2* |  |  |
| *CLN3* |  | *GCSH* |  | *MECP2* |  | *QARS* |  | *ST3GAL3* |  |  |
| *CLN5* |  | *GLDC* |  | *MEF2C* |  | *RANBP2* |  | *ST3GAL5* |  |  |
| *CLN6* |  | *GLRA1* |  | *MFSD8* |  | *RBFOX1* |  | *STRADA* |  |  |
| *CLN8* |  | *GNAO1* |  | *MTOR* |  | *RBFOX3* |  | *STX1B* |  |  |
| *GOSR2* |  | *NEDD4L* |  | *NECAP1* |  | *RELN* |  | *STXBP1* |  |  |

**Supplementary Table 2:** Variants of uncertain significance in established and candidate epilepsy genes.

| **Case** | **Gene** | **Transcript Reference** | **OMIM**  **Phenotype** | **Variant Type** | **AA change^a^** | **cDNA Variant^a^** | **CADD Phred** | **Polyphen-2 Scores^b^** | **Post-Neonatal Epilepsy** |
| --- | --- | --- | --- | --- | --- | --- | --- | --- | --- |
| 5 | *CHRNA2* | ENST00000240132 | ADNFLE 4 (AD) | Inherited (mat) | p.D469E | c.1407T>A | 22.2 | 0.88 / 0.74 | Yes |
| 5 | *CACNA1H* | ENST00000565831 | Epilepsy Susceptibility | Inherited (mat) | p.P618L | c.1853C>T | 25.0 | 0.89 / 0.53 | Yes |
| 7 | *RYR3* | ENST00000389232 | None | Inherited (pat) | p.G592R | c.1774G>A | 32.0 | 1.0 / 1.0 | Yes |
| 11 | *IQSEC2* | ENST00000375368 | X-linked mental retardation 1/78 (XL) | *de novo*  *(compound)* | p.G84S  p.A186S | c.250G>A  c.556G>T | 21  20.4 | 0.0 / 0.0  0.0 / 0.02 | No |
| 12 | *PCDH19­* | ENST0000025553 | EIEE 9 (XL) | Inherited (pat) | p.D960N | c.2878G>A | 24.9 | 0.87 / 0.32 | No |

^a^HGVS notation.

^b^Polphen-2 scores presented as HumDiv / HumVar.

Abbreviations: AA = amino acid; AD = autosomal dominant; ADNFLE = autosomal dominant nocturnal frontal lobe epilepsy; EIEE = early infantile epileptic encephalopathy; mat = maternal; pat = paternal; XL = X-linked.

**Supplementary Table 3:** 'Pathogenic' and 'likely pathogenic' variants in coronary artery disease genes

| **Case** | **Gene** | **Transcript Reference** | **Inheritance** | **Variant Type** | **Class** | **AA change^a^** | **cDNA Variant^a^** | **CADD Phred** | **Polyphen-2 Scores^b^** | **Post-Neonatal Epilepsy** |
| --- | --- | --- | --- | --- | --- | --- | --- | --- | --- | --- |
| 2 | *AKAP9* | ENST00000359028 | AD | Inherited (pat) | Missense | p.E963Q | c.2887G>C | 24.4 | 1.0 / 0.92 | Yes |
| 11 | *FHL1* | ENST00000370676 | XL | *de novo* | Splice/  deletion | - | c.549+2_549+3del | - | - | No |

^a^HGVS notation and GRCh37 genome reference.

^b^Polphen-2 scores presented as HumDiv / HumVar.

Abbreviations: AA = amino acid; AD = autosomal dominant; mat = maternal; pat = paternal; XL = X-linked.

**Supplementary Table 4:** Non-inherited "pathogenic" and "likely pathogenic" variants in epilepsy or coronary artery disease gene sets

| **Gene** | **Transcript Reference** | **Variant Class** | **AA change^a^** | **cDNA Variant^a^** | **CADD Phred** | **Polyphen-2 Scores^b^** | **Child with Post-Neonatal Epilepsy** |
| --- | --- | --- | --- | --- | --- | --- | --- |
| Epilepsy Genes (Established and Candidate) | | | | | | | |
| *GRIN1* | ENST00000371550 | Splice | - | g. 140052978A>T | 21.3 | - | Yes |
| Coronary Artery Disease Genes | | | | | | | |
| *TPM1* | ENST00000317516 | Frameshift | p.D222EfsTer7 | c.666_667del | 30.0 | 1.0 / 0.92 | Yes |

^a^HGVS notation and GRCh37 genome reference.

^b^Polphen-2 scores presented as HumDiv / HumVar.

Abbreviations: AA = amino acid; AD = autosomal dominant; mat = maternal; pat = paternal; XL = X-linked.

**Supplementary Figure 1:** IGV inspection of established and candidate epilepsy genes in the proband and parents.

1. CASK:


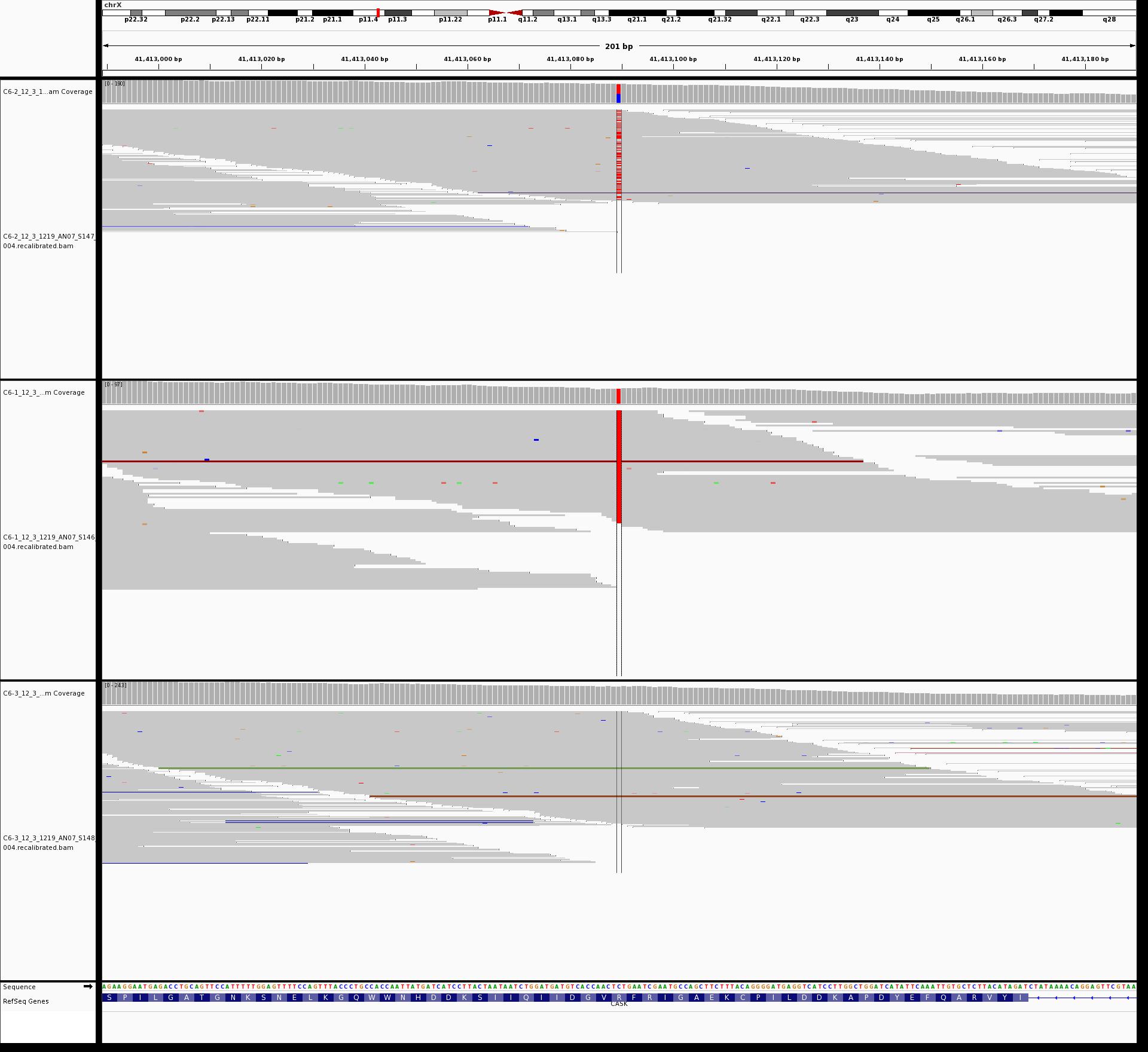


1. CPA6:


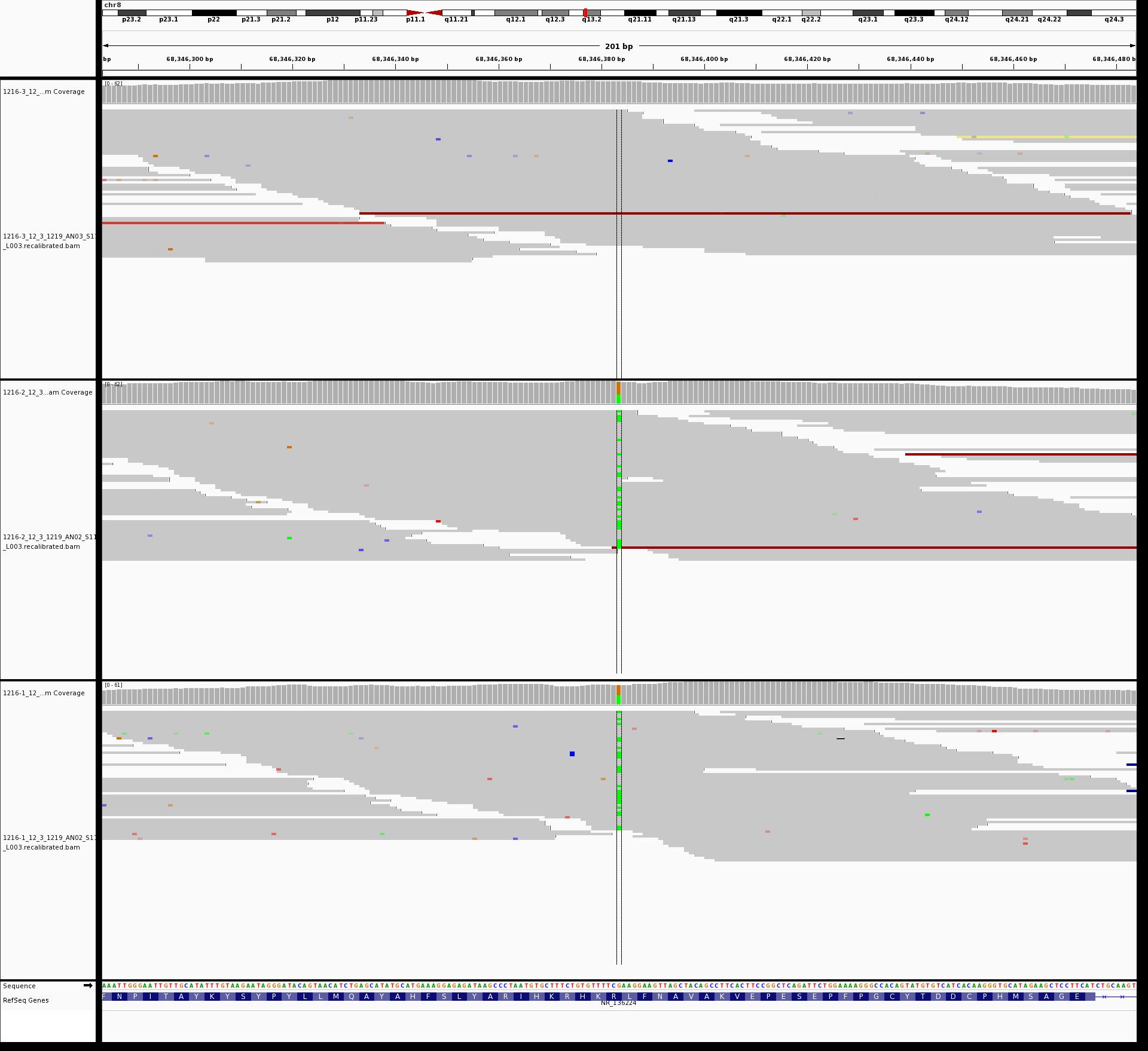


1. KCNT1:
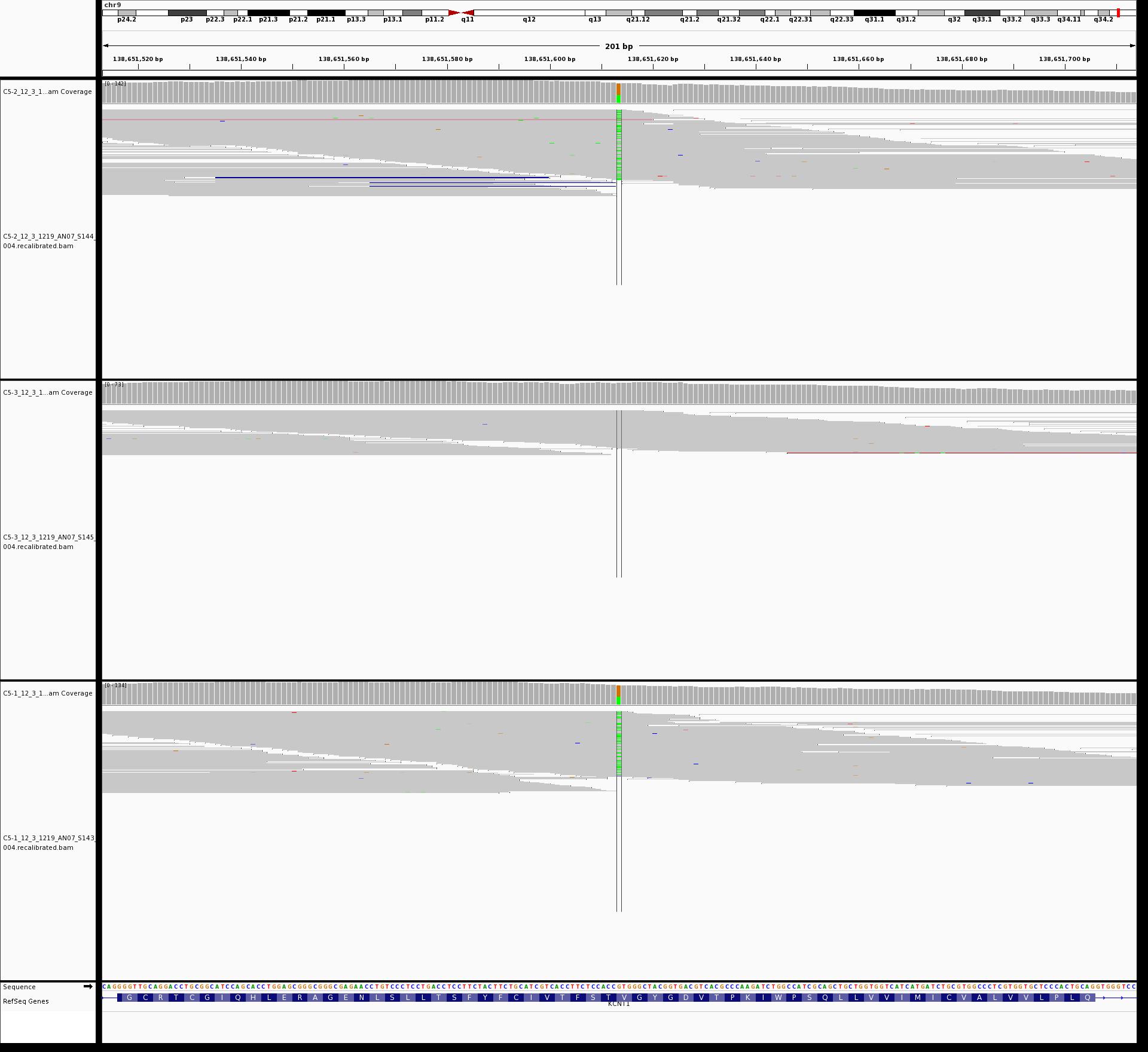

2. MAGI2:


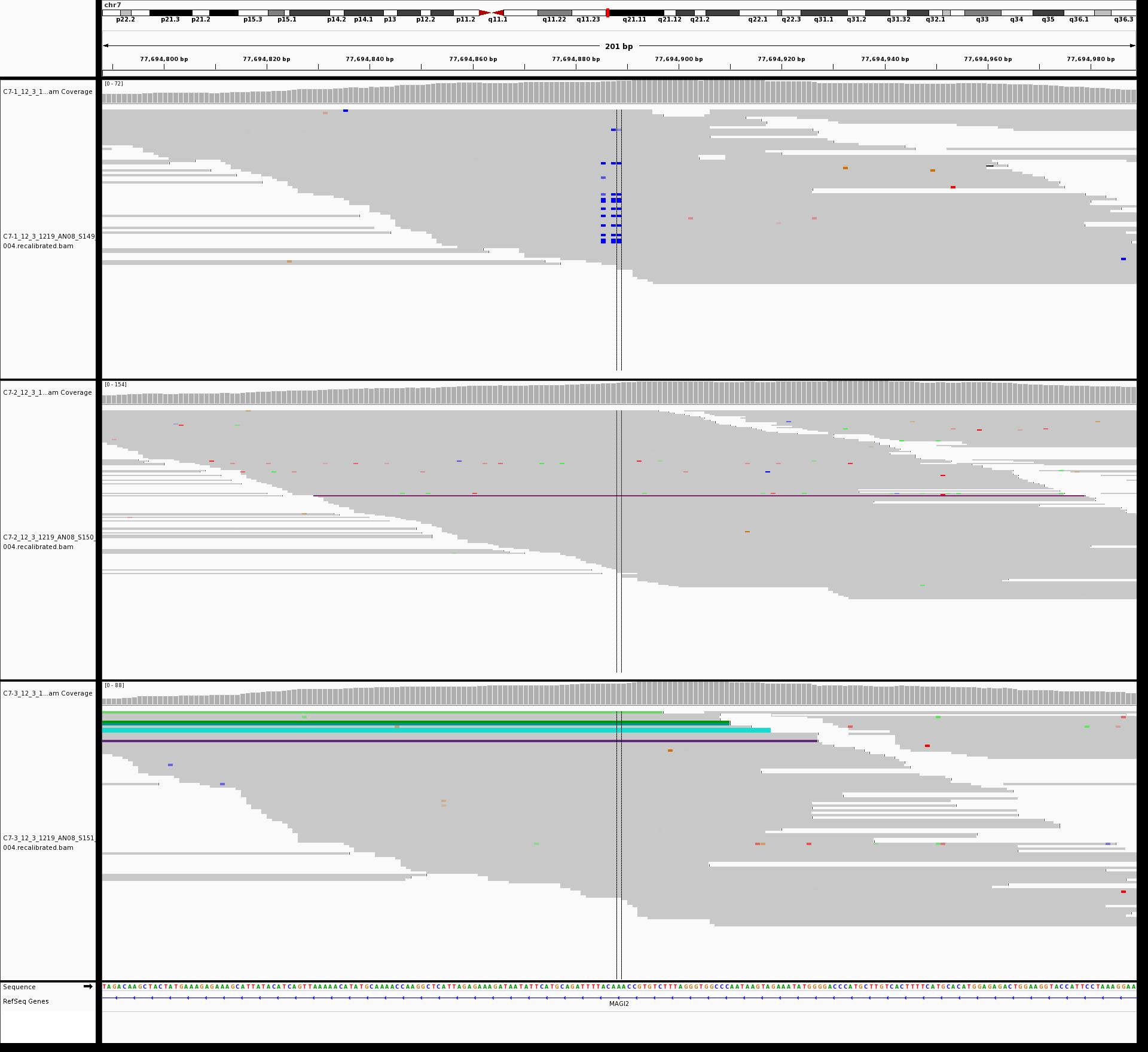


1. PRRT2:
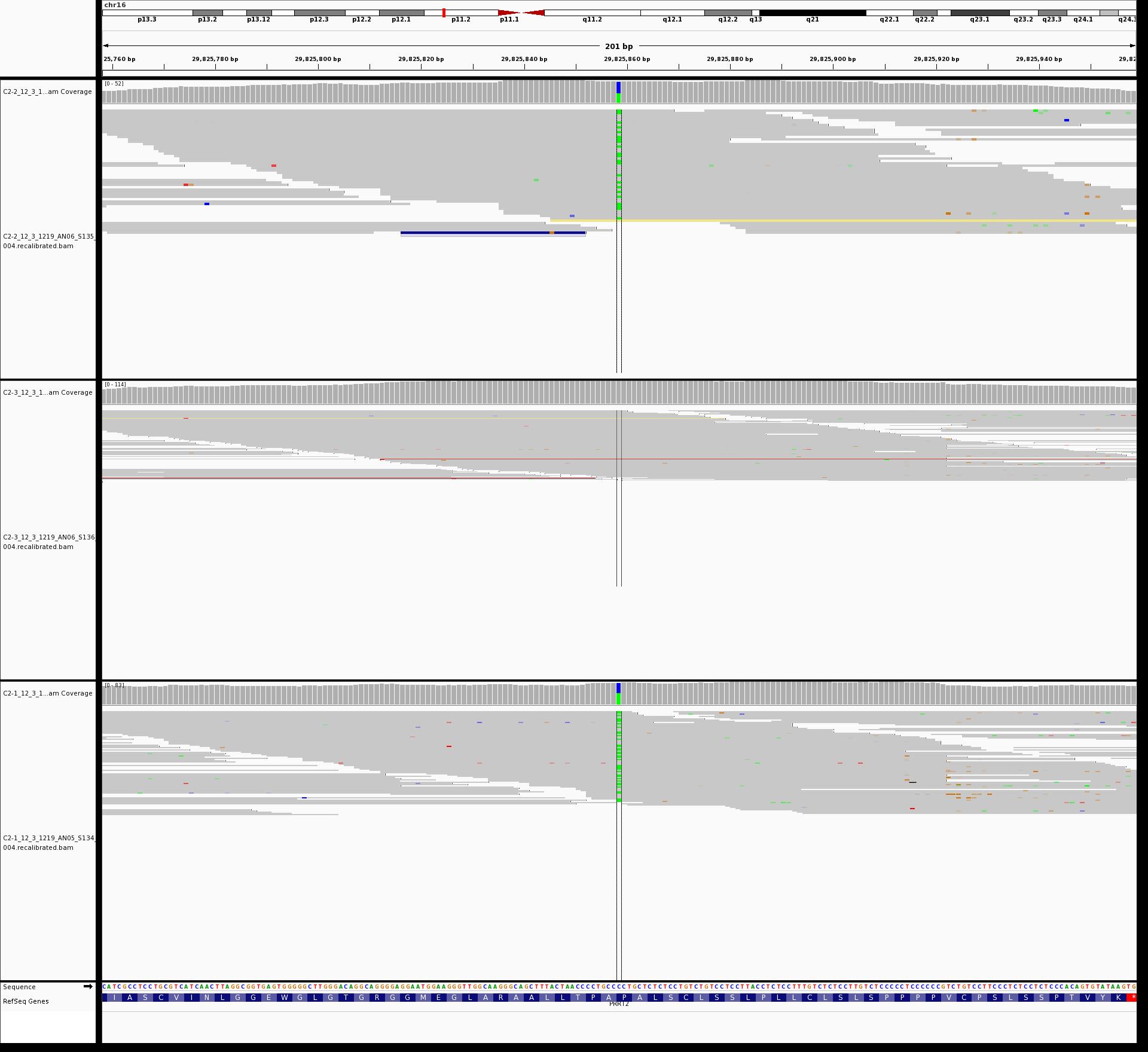

2. RBFOX3:
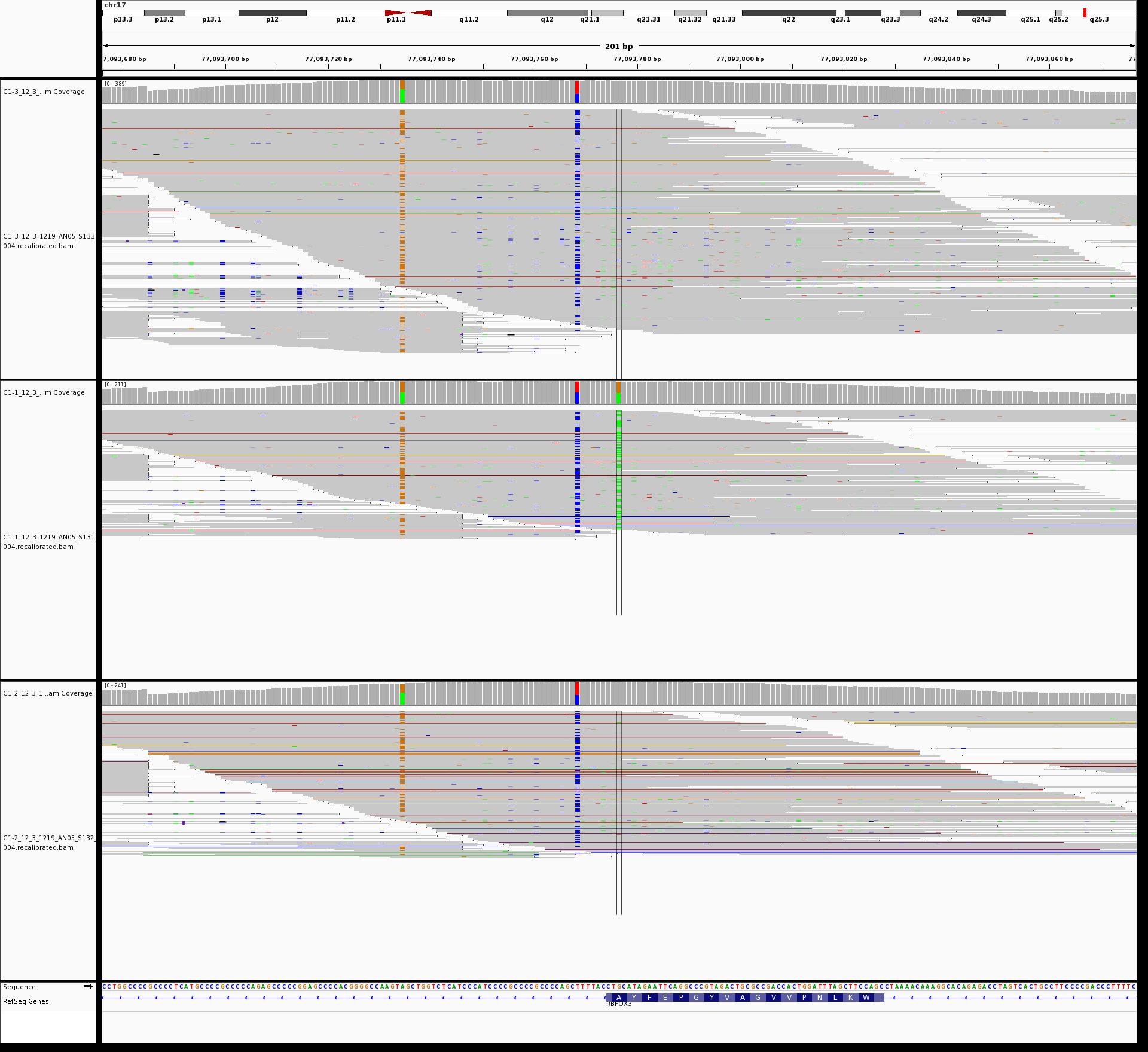


**Supplementary Figure 2:** IGV inspection of *de novo* non-epilepsy gene variants identified on whole exome sequencing in the proband and parents.

1. ATF6B:


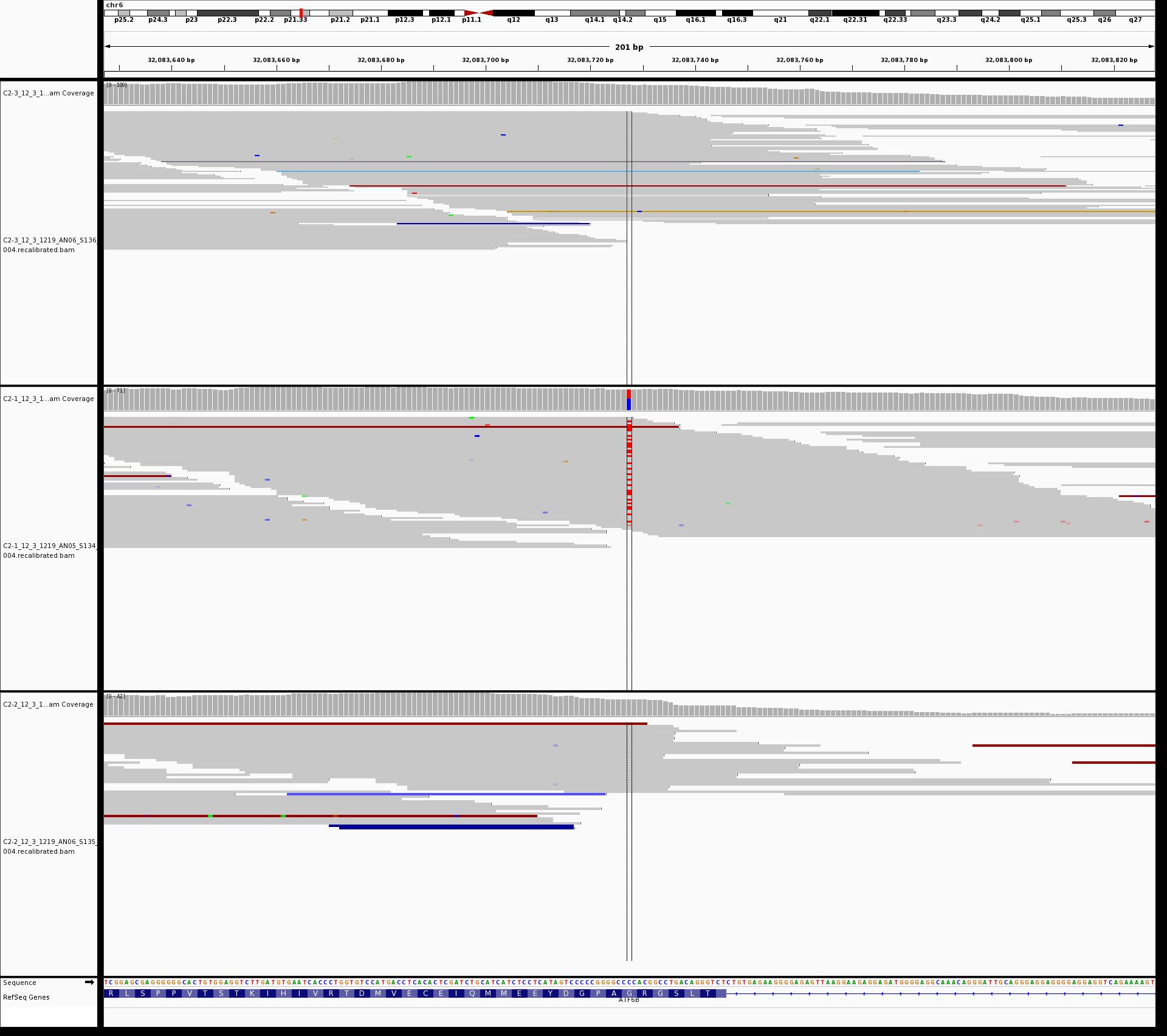


1. CAPN7:
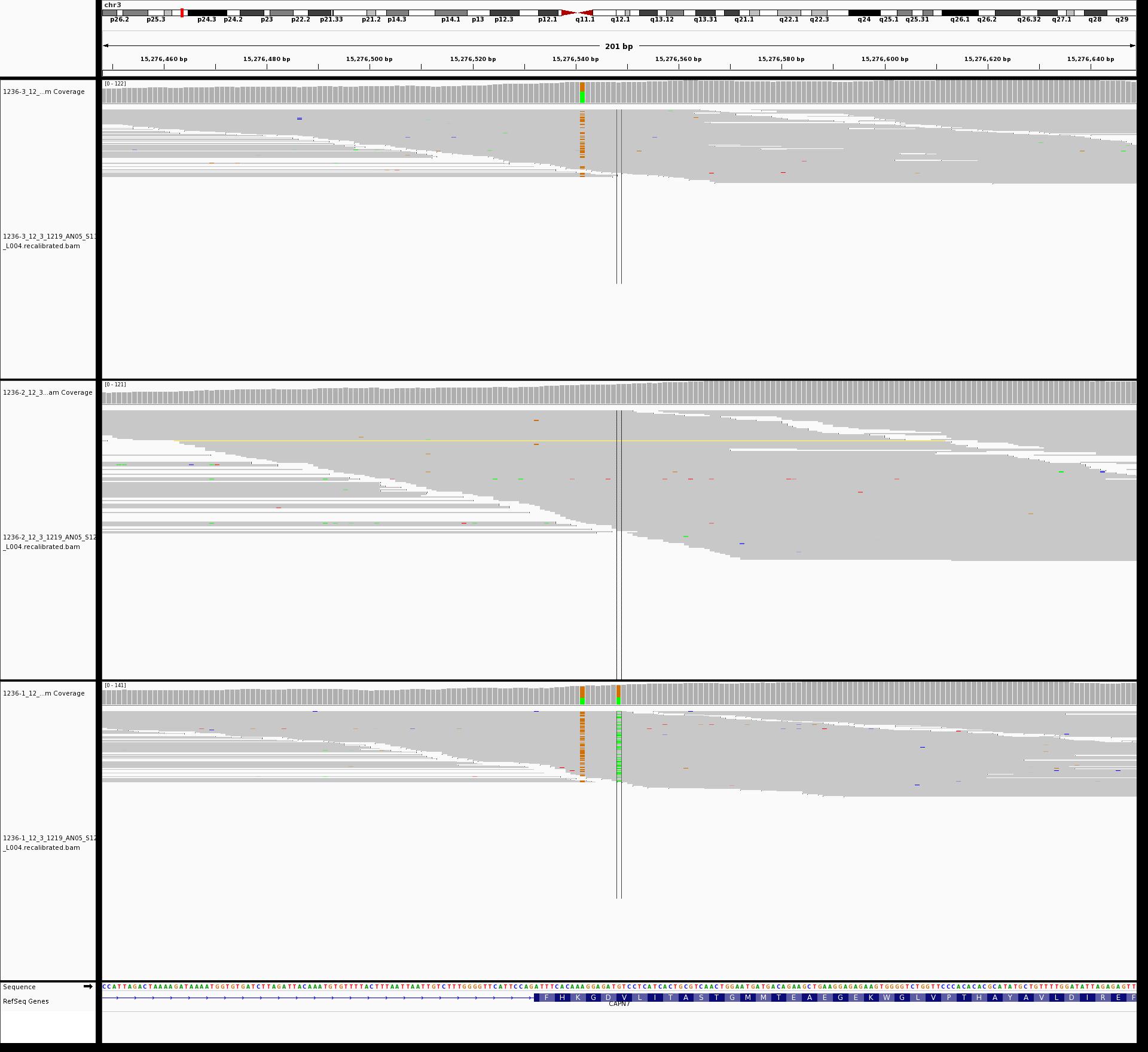

2. CSTF2T:
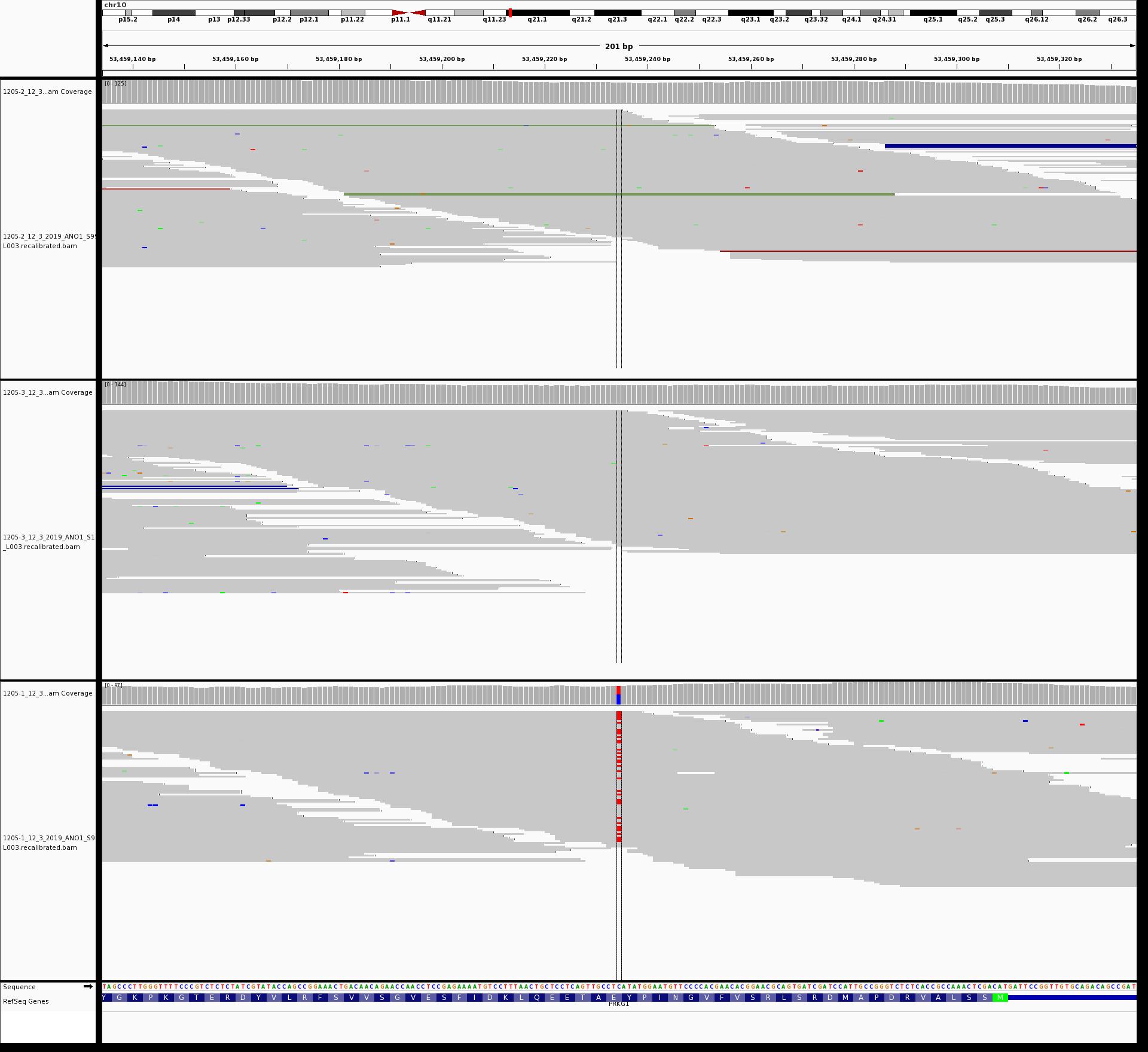

3. DCHS2:
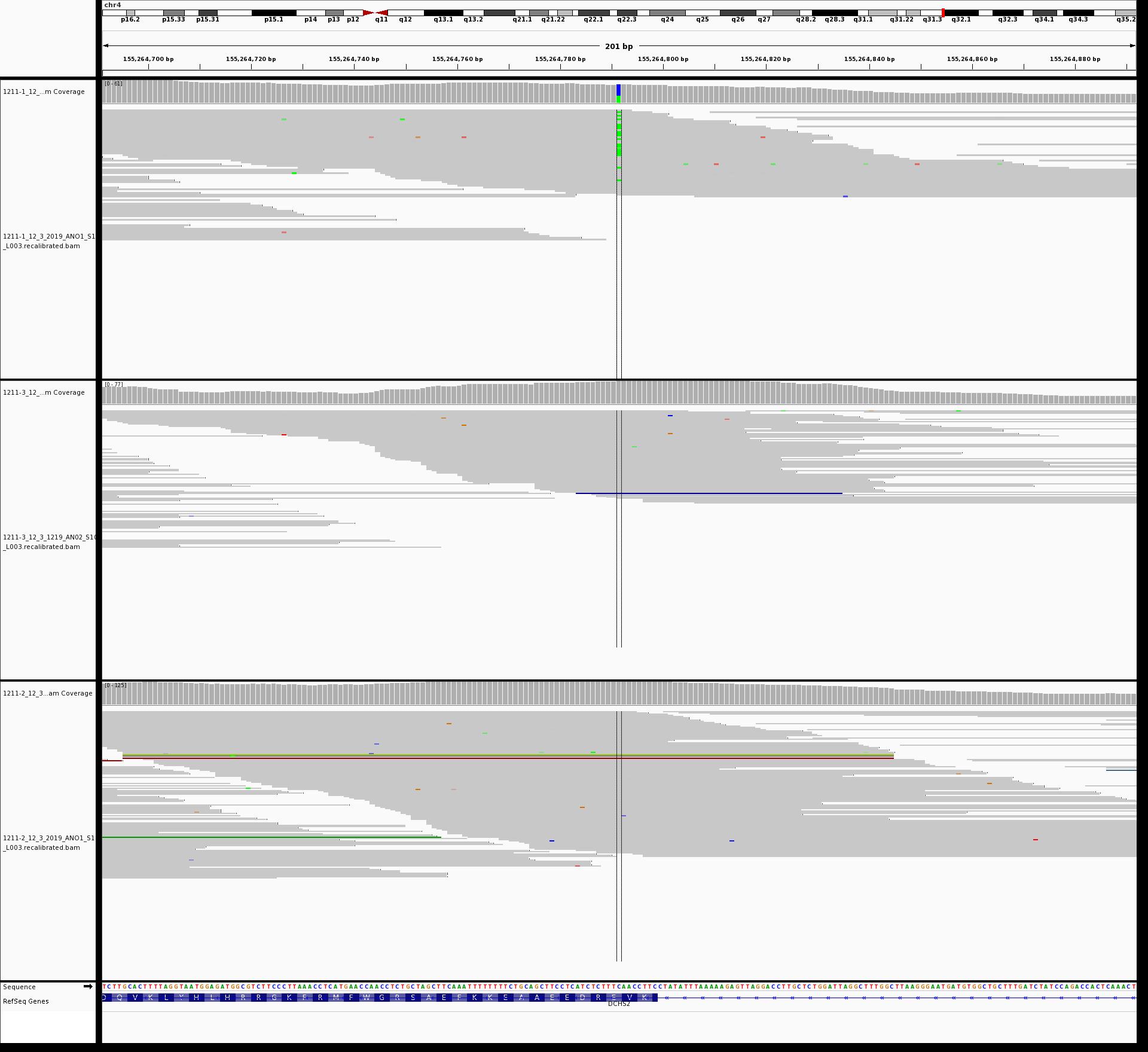

4. DHRS11:
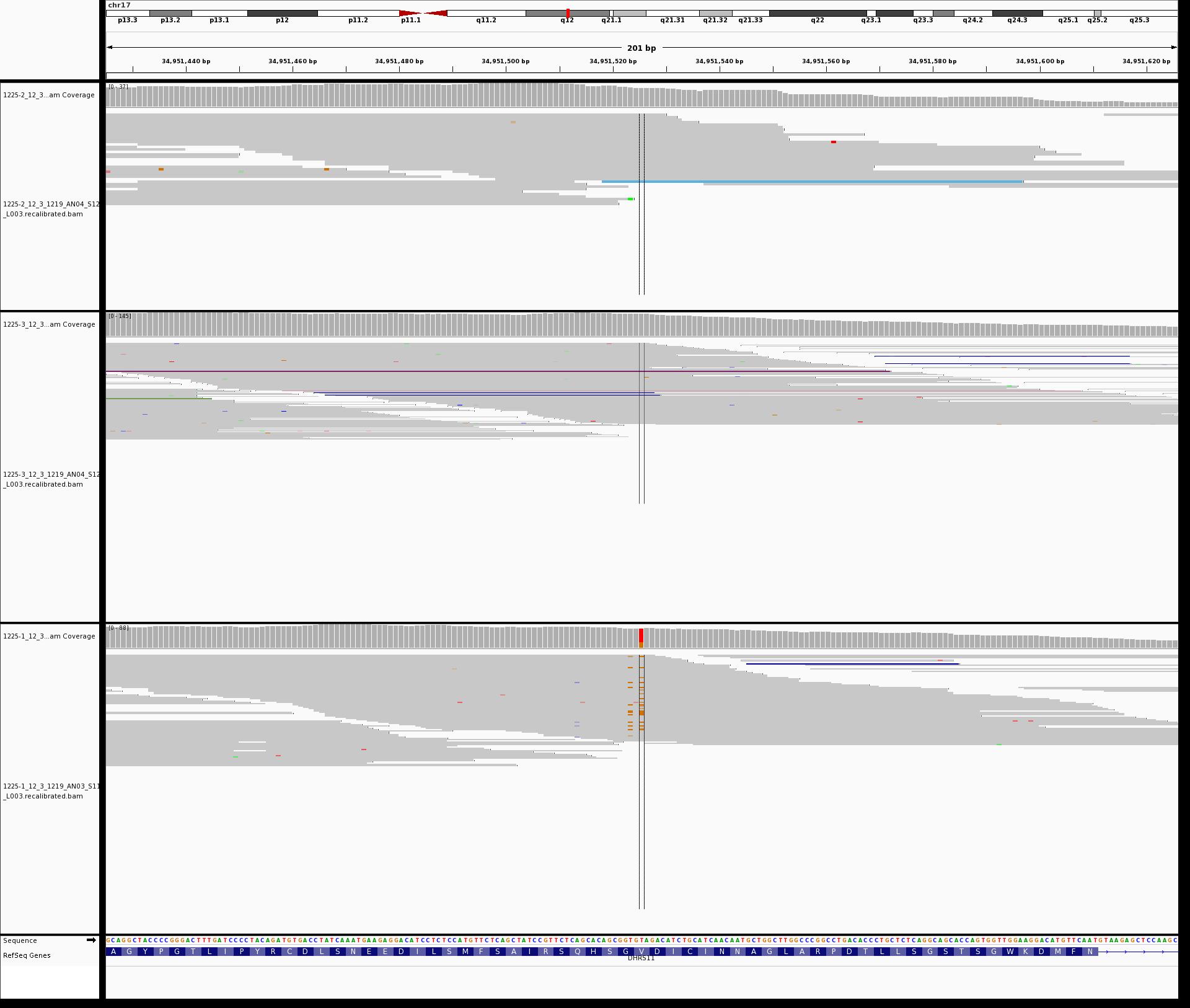

5. ELF1:
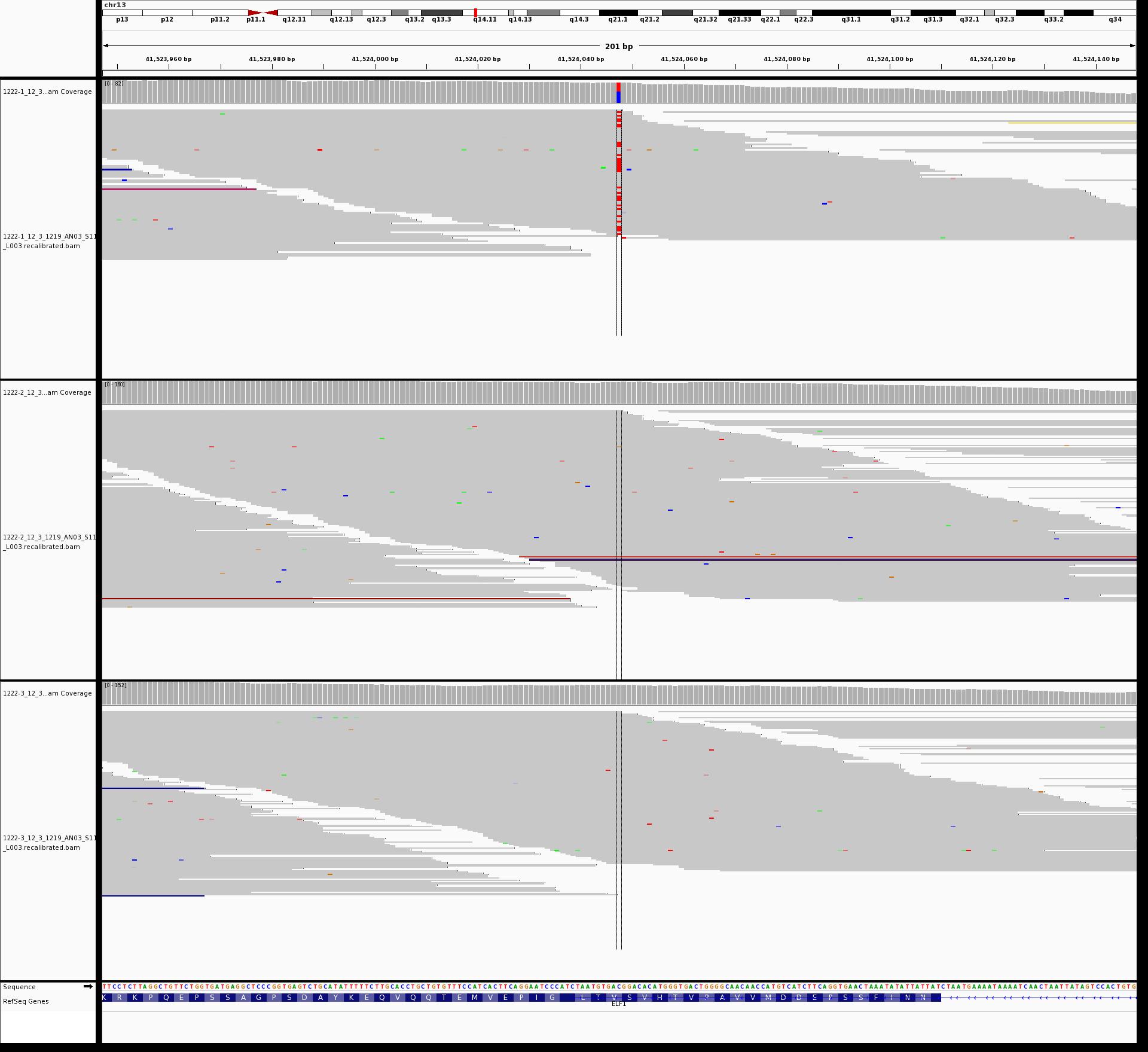

6. ENOX1:
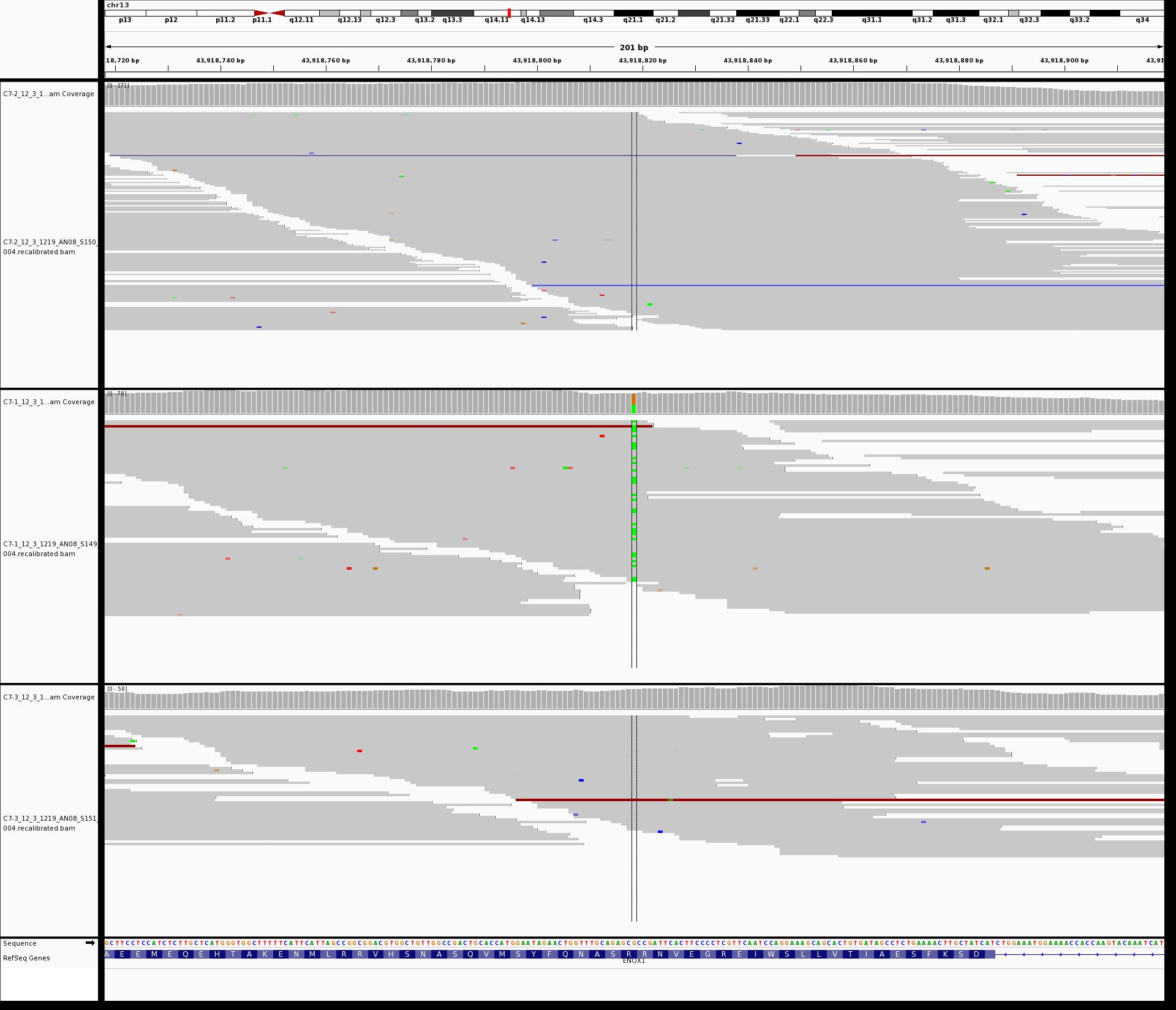

7. FAM186A:
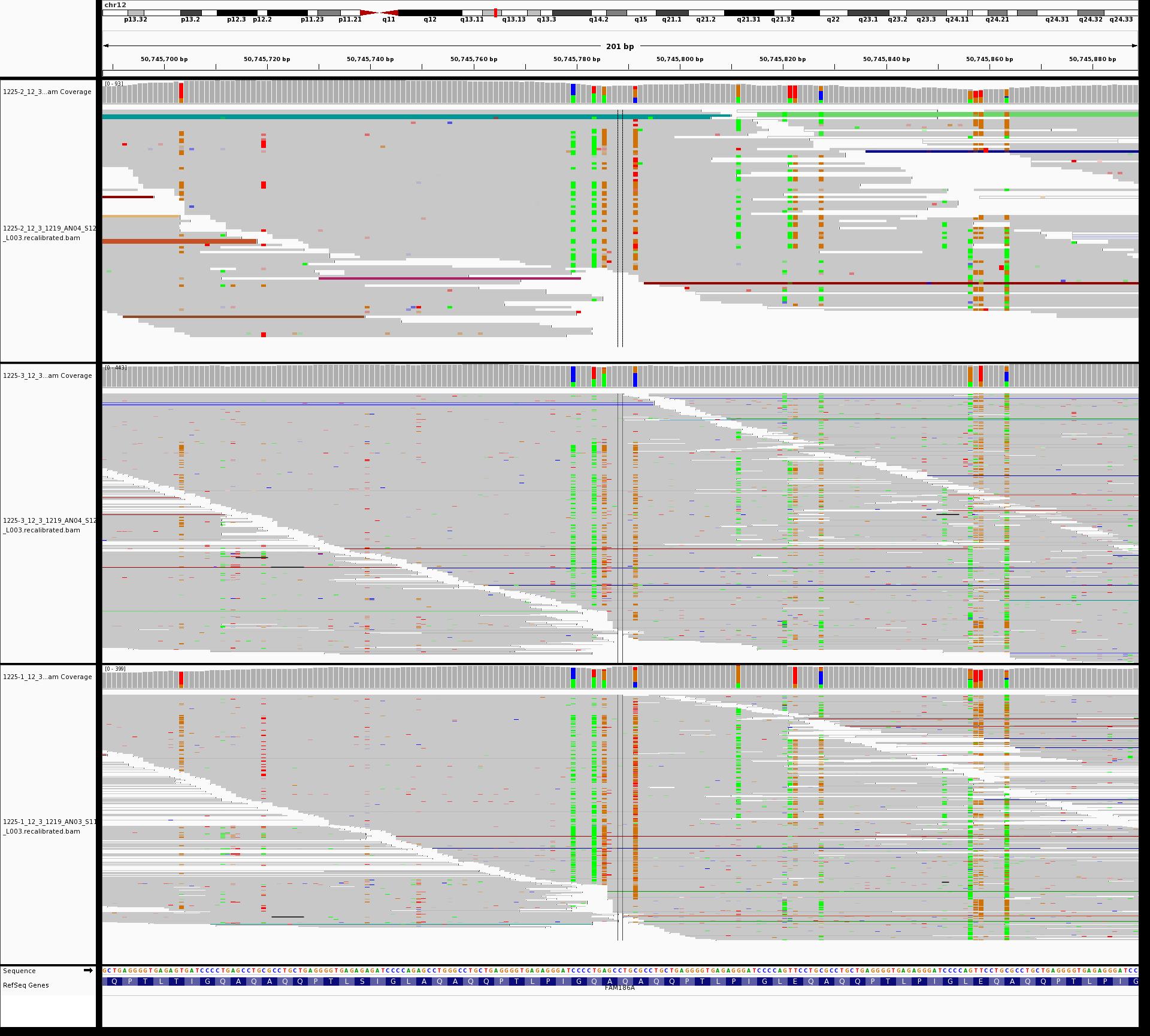

8. GNB3:
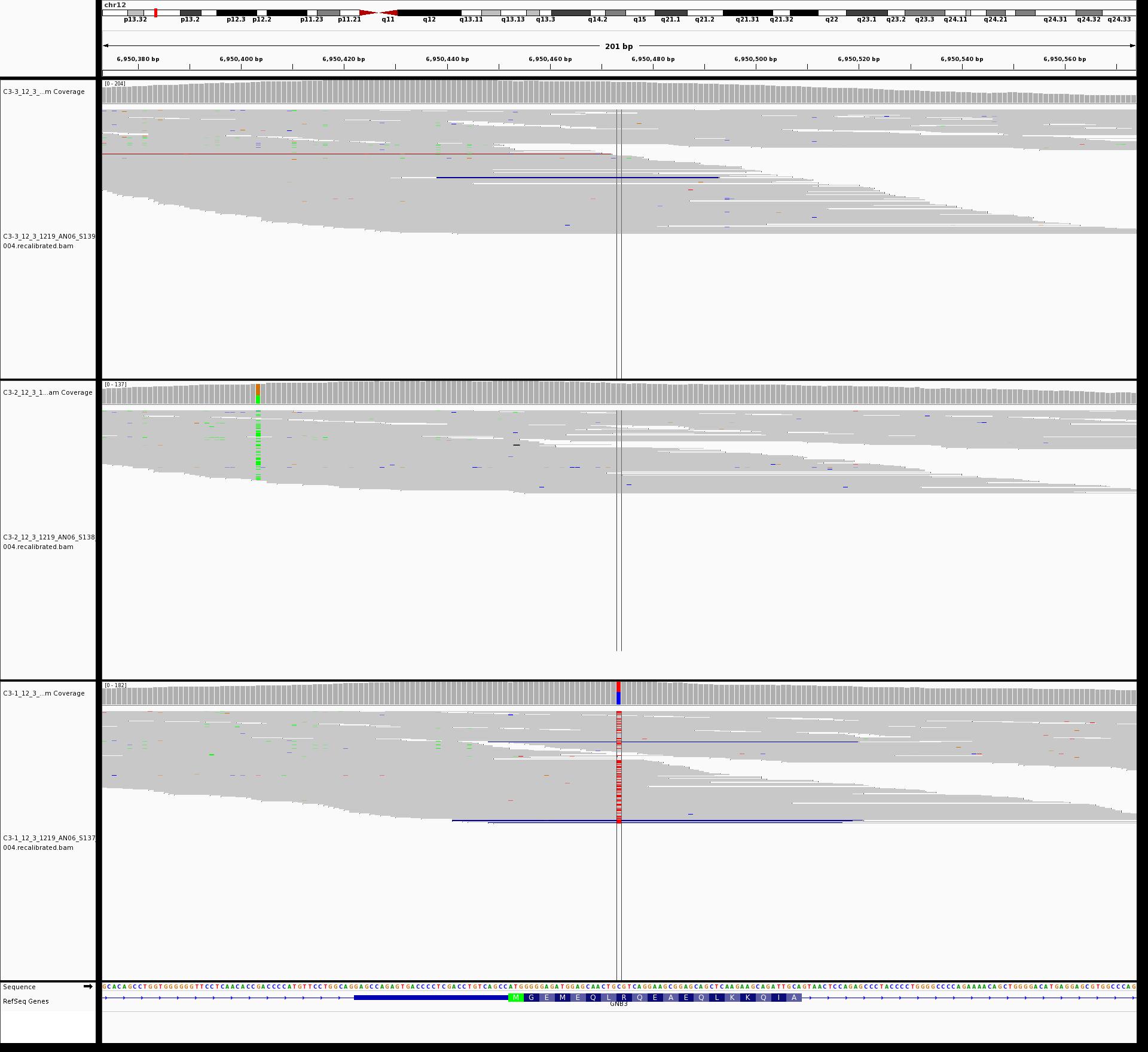

9. HECTD4:
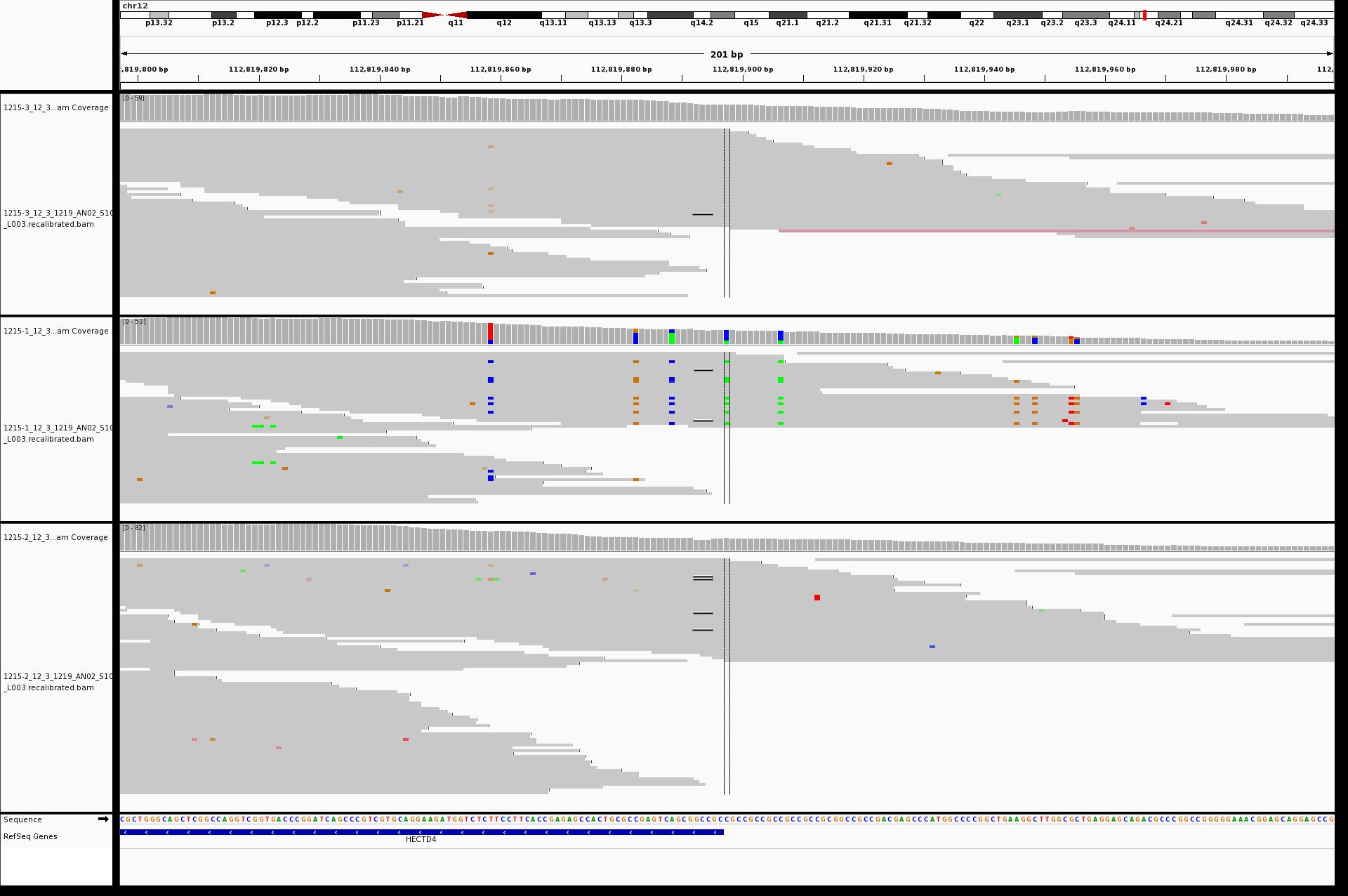

10. MIF4GD:


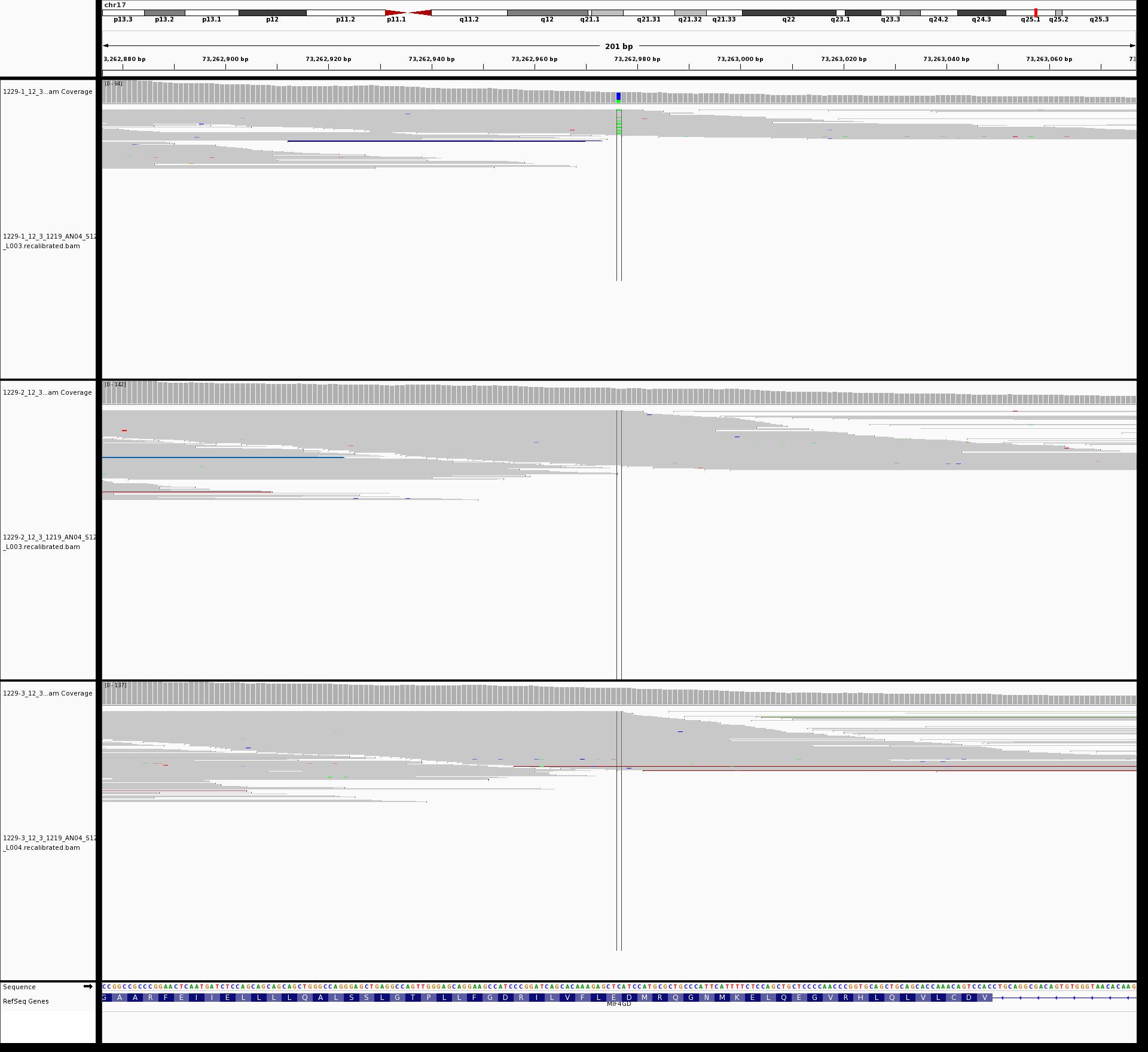


1. OSBP:


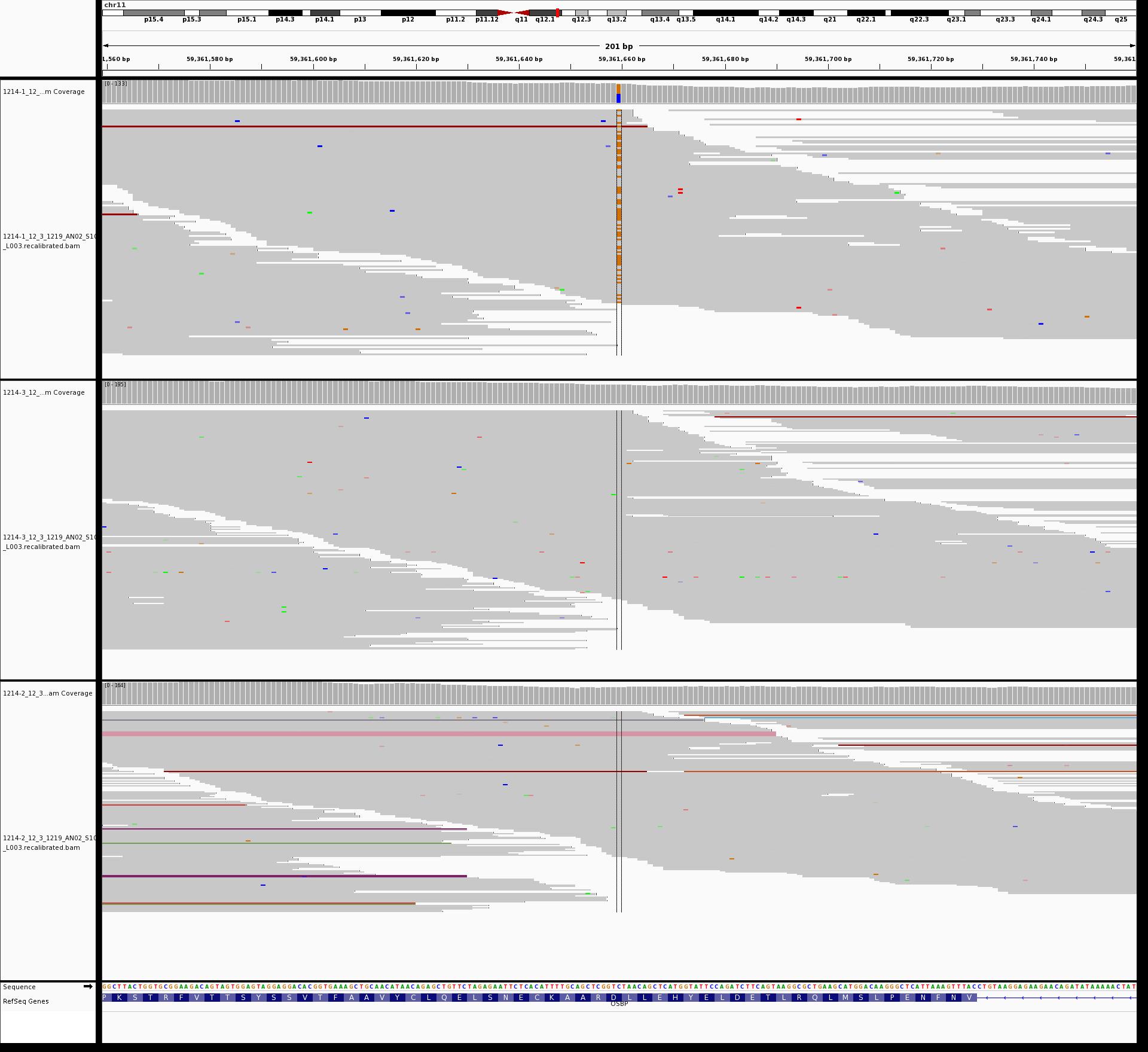


1. PCBP2:
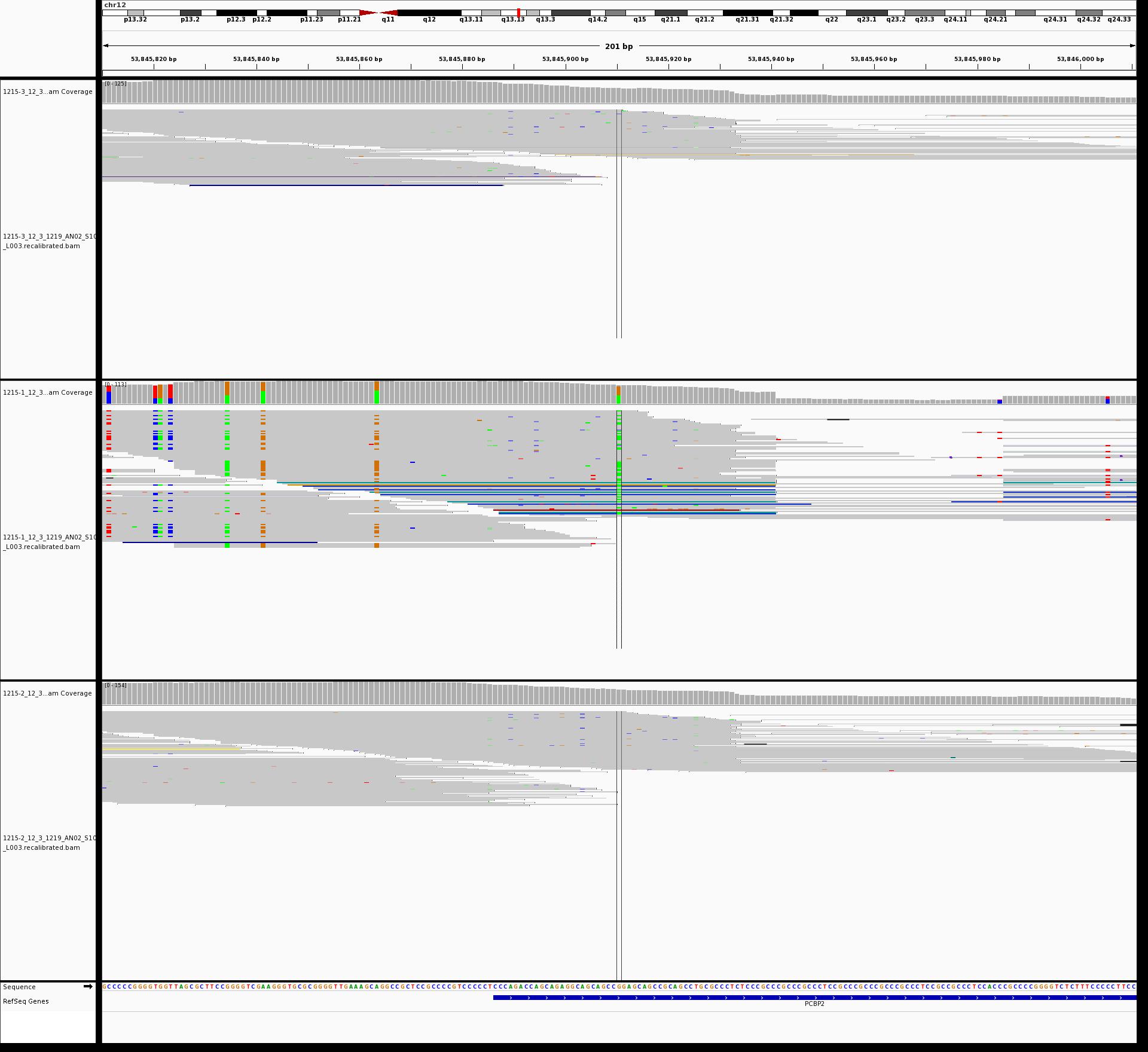

2. PTNP14:
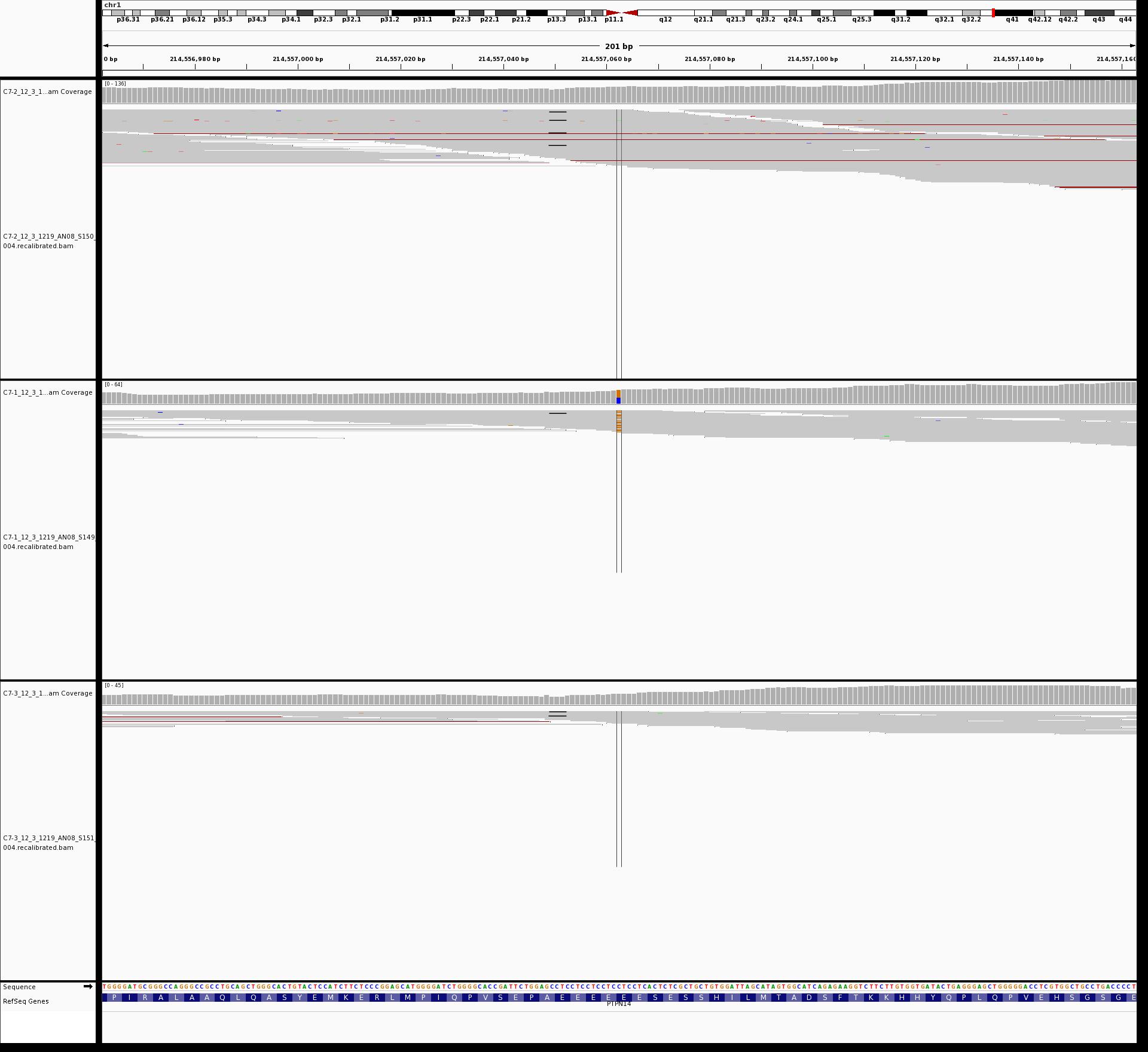

3. SNRPB:
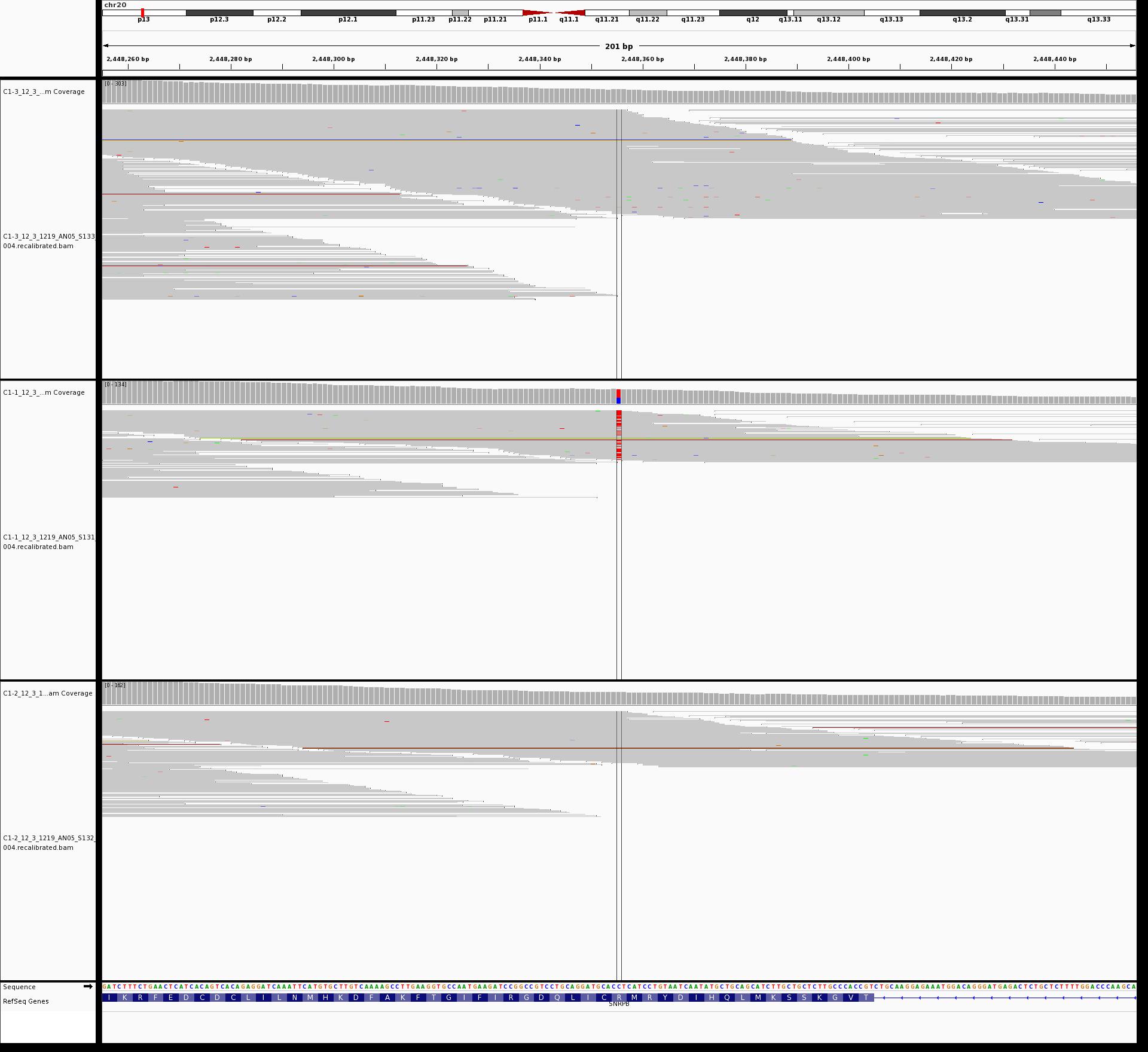

4. UBXN11:
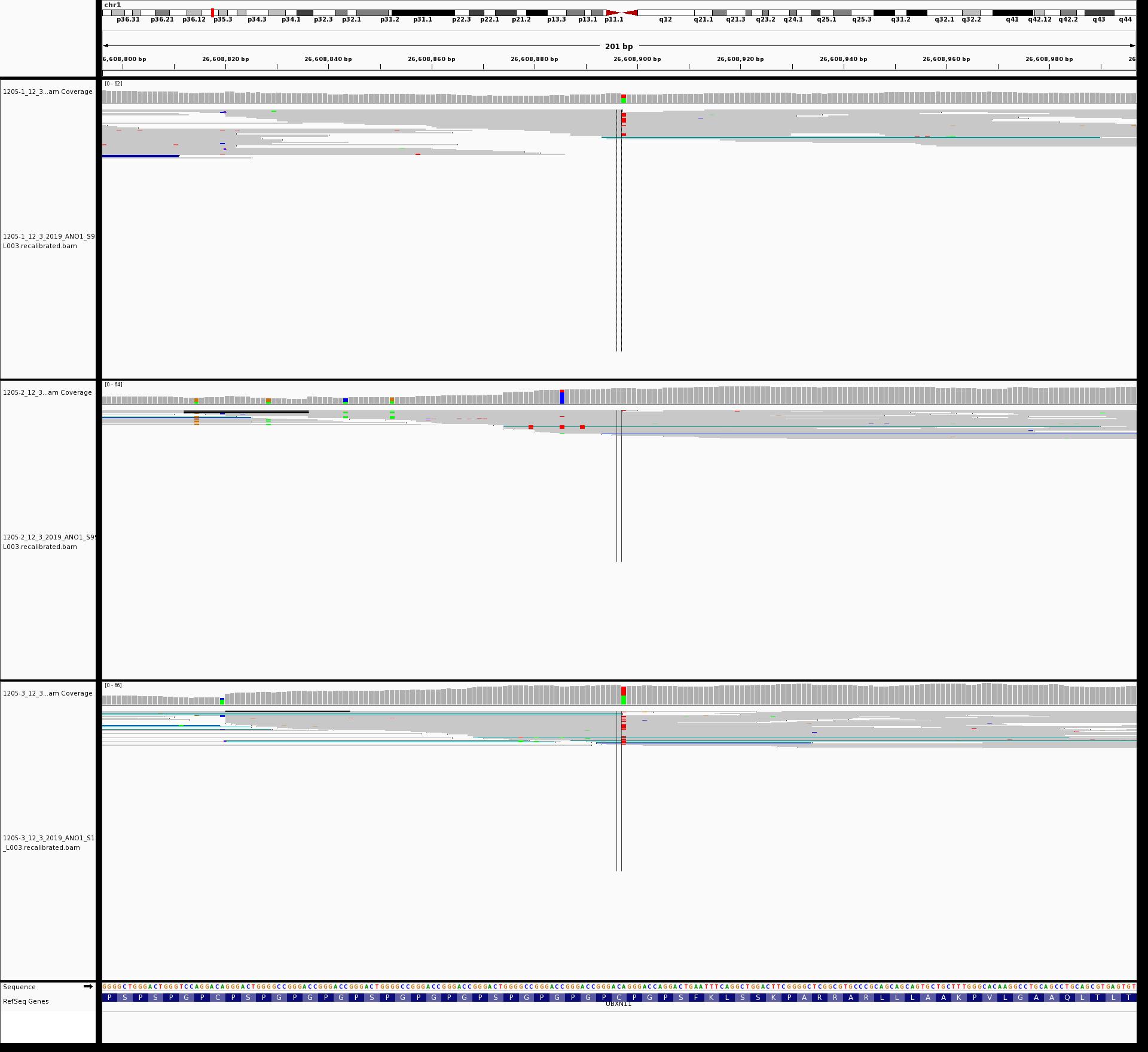

5. VPS37A:
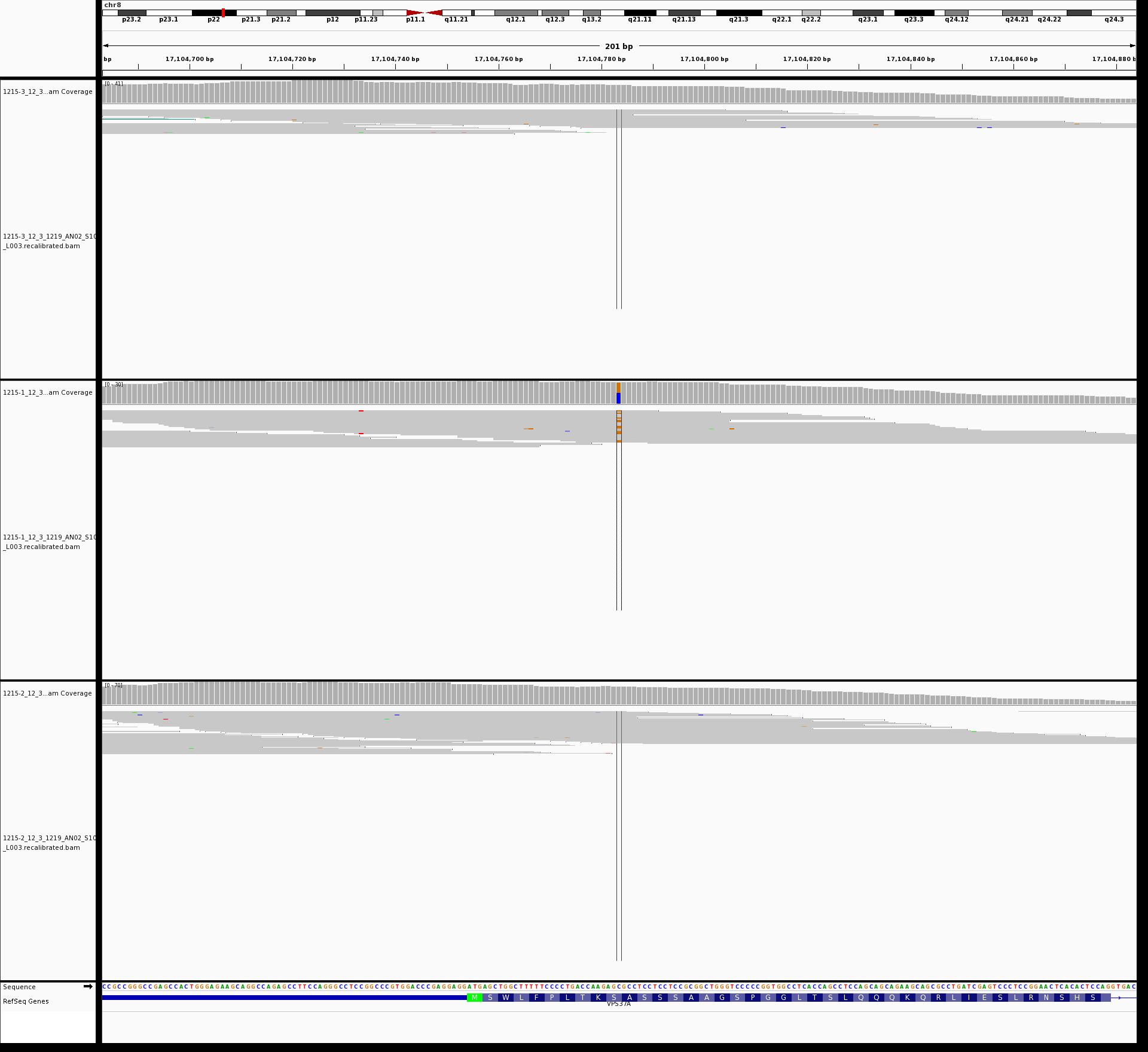

Supplement: Supplementary file 1 — Supplementary Materials [file 41390_2021_1509_MOESM1_ESM.docx]
